# The Role of GABA in the Dorsal Striatum-Raphe Nucleus Circuit Regulating Stress Vulnerability in Male Mice with High Levels of Shati/Nat8l

**DOI:** 10.1523/ENEURO.0162-23.2023

**Published:** 2023-10-19

**Authors:** Hajime Miyanishi, Shiori Suga, Kazuyuki Sumi, Miho Takakuwa, Naotaka Izuo, Takashi Asano, Shin-ichi Muramatsu, Atsumi Nitta

**Affiliations:** 1 Department of Pharmaceutical Therapy and Neuropharmacology, Faculty of Pharmaceutical Sciences, Graduate School of Medicine and Pharmaceutical Sciences, University of Toyama, Toyama 930-0194, Japan; 2 Division of Neurological Gene Therapy, Center for Open Innovation, Jichi Medical University, Shimotsuke 329-0498, Japan; 3 Center for Gene & Cell Therapy, The Institute of Medical Science, The University of Tokyo, Tokyo 108-0071, Japan

**Keywords:** 5-hydroxytryptamine, dorsal raphe nucleus, dorsal striatum, Shati/Nat8l, stress, stress sensitivity

## Abstract

Depression is a frequent and serious illness, and stress is considered the main risk factor for its onset. First-line antidepressants increase serotonin (5-hydroxytryptamine; 5-HT) levels in the brain. We previously reported that an *N*-acetyltransferase, Shati/Nat8l, is upregulated in the dorsal striatum (dSTR) of stress-susceptible mice exposed to repeated social defeat stress (RSDS) and that dSTR Shati/Nat8l overexpression in mice (dSTR-Shati OE) induces stress vulnerability and local reduction in 5-HT content. Male mice were used in this study, and we found that dSTR 5-HT content decreased in stress-susceptible but not in resilient mice. Moreover, vulnerability to stress in dSTR-Shati OE mice was suppressed by the activation of serotonergic neurons projecting from the dorsal raphe nucleus (dRN) to the dSTR, followed by upregulation of 5-HT content in the dSTR using designer receptors exclusively activated by designer drugs (DREADD). We evaluated the role of GABA in modulating the serotonergic system in the dRN. Stress-susceptible after RSDS and dSTR-Shati OE mice exhibited an increase in dRN GABA content. Furthermore, dRN GABA content was correlated with stress sensitivity. We found that the blockade of GABA signaling in the dRN suppressed stress susceptibility in dSTR-Shati OE mice. In conclusion, we propose that dSTR 5-HT and dRN GABA, controlled by striatal Shati/Nat8l via the dSTR-dRN neuronal circuitry, critically regulate stress sensitivity. Our study provides insights into the neural processes that underlie stress and suggests that dSTR Shati/Nat8l could be a novel therapeutic target for drugs against depression, allowing direct control of the dRN serotonergic system.

## Significance Statement

Given that 30% of depression patients have resistant to conventional antidepressants, finding novel therapeutic strategies for its disease is required. We previously demonstrated that the overexpression of Shati/Nat8l, *N*-acetyltransferase, in the dorsal striatum (dSTR) of mice induces stress vulnerability. dSTR 5-HT (5-hydroxytryptamine) levels are downregulated in stress-susceptible, nonresilient mice exposed to repeated social defeat stress (RSDS). Stress vulnerability in dSTR Shati/Nat8l overexpression mice was suppressed by the activation of serotonergic neurons projecting from the dorsal raphe nucleus (dRN) to the dSTR. We discovered that dRN GABA content correlated with stress sensitivity and inhibited GABA signaling in dRN-induced stress resilience. We suggest that novel bidirectional dSTR-dRN circuits determine the stress sensitivity underlying depression pathology.

## Introduction

Mood disorders, including bipolar disorder and depressive disorders (depression), are frequent and serious illnesses. Between 1990 and 2019, the number of patients with depression increased by 64.4% (GBD 2019 Mental Disorders Collaborators, 2022). Its characteristics include a high prevalence, a lifetime prevalence of 16%, and resistance to treatment (Kessler et al., 2003; Mata et al., 2015; Pandarakalam, 2018). One in ten people has a decreased quality of life because of a wide array of depressive symptoms, such as emotional suffering, cognitive dysfunction, and social impairment (Hauenstein, 2003). Moreover, ∼30–50% of patients have forms of treatment resistance to conventional antidepressant drugs (Dudek et al., 2021). Therefore, novel therapeutic strategies and targets for drugs to treat depression are required. However, numerous unclear aspects of depression pathogenesis have prevented progress in this field, particularly in providing effective therapies for refractory forms of the disease.

Stress is linked to depressive pathology, and some studies have indicated that stressful life events have a substantial causal relationship with depression onset (Kendler et al., 1999). However, this is not always the case because some individuals are resilient (Fleshner et al., 2011). Mice can also be categorized into two groups: stress-susceptible mice, showing depression-like behaviors, and stress-resilient mice, showing no behavioral changes despite being exposed to the same stress conditions (Golden et al., 2011). Controlling stress resilience could lead to the development of new antidepressants. However, the regulatory mechanisms of stress sensitivity have not been elucidated.

An imbalance in serotonin (5-hydroxytryptamine; 5-HT) levels is associated with the pathogenesis of depression (Yuan et al., 2015; Dell'Osso et al., 2016). Over the last 50 years, 5-HT deficits have been targeted in the treatment of depression, and selective serotonin reuptake inhibitors (SSRIs) have been used as first-line antidepressants (Hofmann et al., 2017). Stress also influences 5-HT neurotransmission (Graeff et al., 1996; Hale et al., 2012). Alterations in brain 5-HT, 5-HT-related molecular content (Kang et al., 2005), and serotonergic activity (Paul et al., 2011; J. Zhang et al., 2012), have been observed in individuals exposed to acute or chronic stress. In behavioral experiments, mice with hereditary central 5-HT deficiency are more susceptible to social stress (Sachs et al., 2015), and increasing 5-HT levels with SSRIs prevents the impairment of stress adaptation (Uno et al., 2019). In addition, 5-HT is predominantly synthesized in the raphe nucleus by tryptophan hydroxylase (TPH; Ishimura et al., 1988). In the raphe nucleus of both rodents and humans, almost all serotonergic cell bodies are located in the dorsal region (dRN; Ishimura et al., 1988; Hornung, 2003).

We previously reported that dorsal striatum (dSTR)-specific overexpression of *N*-acetyltransferase Shati/Nat8l in mice induces a decrease in dSTR 5-HT levels (Miyamoto et al., 2017). Although 50% of control mice exposed to repeated social defeat stress (RSDS) showed a depressive phenotype, almost all dSTR Shati/Nat8l overexpression in mice (dSTR-Shati OE) showed depression-like behaviors. Furthermore, we demonstrated that Shati/Nat8l overexpression mice were vulnerable to subthreshold social stress (Uno et al., 2019). Conversely, the knock-down of striatal Shati/Nat8l induces stress resilience (Miyanishi et al., 2021). Furthermore, we suggest that Shati/Nat8l could be used as a biomarker for diagnosing depression in a clinical study (Miyanishi et al., 2020). Shati/Nat8l was previously isolated from the brains of animals with induced psychosis (Niwa et al., 2007), and exhibited *N-acetyl* transfer activity, catalyzing the synthesis of *N*-acetylaspartate (NAA) from acetyl-CoA and aspartate (Ariyannur et al., 2010). Although the expression of Shati/Nat8l in the dSTR may determine sensitivity to stress, the detailed neural mechanisms underlying this effect have not been demonstrated. Considering the suggestion that Shati/Nat8l in the dSTR contributes to stress sensitivity via the serotonergic system, we focused on the involvement of the dRN.

## Materials and Methods

### Animals

Male C57BL/6J (eight-week-old) and male ICR (four to five months of age) were purchased from Nihon SLC. All mice were housed at constant temperatures (25 ± 1°C) and humidity (50 ± 5%), on a 12/12 h light/dark cycle (lights were turned on at 7 A.M. and turned off at 7 P.M.), with free access to pellets and water. The experimental procedures were approved by the Committee for Animal Experiments of the University of Toyama (approval no. 2021PHA-20) and performed following the National Institutes of Health *Guidelines for the Care and Use of Laboratory Animals*.

### Preparation of virus vectors and microinjection

Adeno-associated virus (AAV) vectors were prepared as described previously (Krzyzosiak et al., 2010; Iida et al., 2013). The AAV-CMV-Shati/Nat8l or Mock vector (AAV2/9) contained the cytomegalovirus (CMV) promoter and cDNA encoding either the 3–6× His-tagged Shati/Nat8l or EGFP sequence, respectively. AAV-CMV-Shati/Nat8l or Mock vectors were microinjected into the dSTR, and AAV-CMV-mock vectors were used as a control for AAV-CMV-Shati/Nat8l. The AAV-pet1-hM3Dq and mock vectors (AAV2/rh10) were provided by Mieda (Kanazawa University, Kanazawa, Ishikawa, Japan; Hasegawa et al., 2014). The AAV-pet1-hM3Dq and mock vectors contained the pet1 promoter and cDNA encoding either the hM3Dq or ChR2::EYFP sequence, respectively. AAV-pet1-hM3Dq or Mock vector was microinjected into the dRN, and AAV-pet1-Mock vectors were used as controls for AAV-pet1-hM3Dq. The titers of the recombinant AAV vectors were as follows: AAV-pet1-hM3Dq, 9.8 × 10^11^; AAV-pet1-Mock (ChR2::EYFP), 5.2 × 10^12^; AAV-CMV- Shati/Nat8l, 1.0 × 10^10^; AAV-CMV-Mock (EGFP), 1.0 × 10^10^ genome copies/ml.

Bilateral microinjection of AAV-CMV-Shati/Nat8l or Mock vector into the dSTR [anteroposterior (AP), 0.5 mm; mediolateral (ML), ±2.0 mm; dorsoventral (DV), 3.0 mm] and of AAV-pet1-hM3Dq or Mock vector into the dRN (AP, −4.4 mm; ML, 0 mm; DV, 3.5 mm) were performed using stereotaxic frame (SR‐5M) following a reference image (Paxinos and Franklin, 2008). This study was approved by the Board of Safety Committee for Recombinant DNA Experiments of the University of Toyama (approval no. G2020PHA‐5).

### Immunostaining

Double immunostaining was performed as described previously (Ibi et al., 2008). Brain tissue fixed with 4% paraformaldehyde (PFA) was sectioned into 30-μm slices using a cryostat (Leica). After slices were permeabilized with 0.25% Triton X‐100 and blocked with 10% goat serum, incubation with primary antibodies against mouse neuronal nuclear antigen (NeuN; 1:500, MBL), rabbit His tag (1:500, MBL), mouse tryptophan hydroxylase (TPH; 1:200, Abcom) and rabbit GFP (1:1000, Sigma-Aldrich) was performed overnight. The slices were washed with Tris-buffered saline containing Tween 20 and incubated with CFTM 594 goat anti-mouse immunoglobulin G (IgG; H+L; 1:1000; Biotium) and CFTM 488 goat anti-rabbit IgG (H+L; 1:1000, Biotium) as secondary antibodies for 2 h. After washing and mounting the sections, immunofluorescence was performed using an AxioCam ICc1 (Carl Zeiss).

### Real-time RT-PCR analysis

Whole brains were placed in a mouse brain matrix (Brain Science Idea) to obtain tissue sections. Total RNA was extracted from these tissues and converted into cDNA using the Prime Script RT Reagent kit (Takara). The mRNA levels were quantified using a Thermal Cycler Dice Real-Time System (Takara) and Thunderbird Syber qPCR Mix (Toyobo). In addition, 36B4 was used as the housekeeping gene. Primer sequences for *Shati/Nat8l* and *36B4* mRNA were as follows (Haddar et al., 2020):

Shati/Nat8l:

forward, 5′-GTGATTCTGGCCTACCTGGA-3′;

reverse, 5′-CCACTGTGTTGTCCTCCTCA-3′;

36B4:

forward, 5′-ACCCTGAAGTGCTCGACATC-3′;

reverse, 5′-AGGAAGGCCTTGACCTTTTC-3′.

### Repeated social defeat stress

ICR mice were used as stressors. Their aggressive behavior was confirmed by screening before the experiments (Golden et al., 2011). C57BL/6J mice were subjected to a 10-min daily physical attack by unfamiliar ICR mice for 10 consecutive days. ICR and C57BL/6J mice were housed in cages separated by a transparent plastic divider, allowing auditory and visual contact for 24 h. The plastic divider was removed during exposure to a physical attack.

### Microdefeat stress

Microdefeat stress was induced as previously described (Golden et al., 2011). On a single day, C57BL/6J mice were subjected to three 5-min physical attacks by ICR mice, with 15 min of rest between each session.

### Behavioral tests

Behavioral tests were performed 24 h after the final defeat session of the RSDS or microdefeat protocol. All tested mice (defeated mice and stress-naive control mice) were subjected to behavioral experiments during light exposure.

#### Social interaction test

The social interaction test was conducted in a plastic open box (40 × 40 × 30 cm) equipped with a mesh cage for the ICR mice (targets). The interaction zone (IZ) was defined as the area surrounding the mesh cage (14 × 24 cm). Avoidance zones (AZs) were defined as the corner areas on opposing sides of the mesh cage (9 × 9 cm). The testing protocol consisted of two 150-s sessions (pre-test or post-test). In the pre-test, the test mice were placed in the center of an open box with no target in the mesh cage, and their activity was recorded for 150 s. After an interval of 30 s, the test mice were returned to the open box, while the target mice were placed in the mesh cage. Their approach or avoidance activity toward the targets was recorded again for 150 s as a post-test. Social interaction ability was assessed by measuring the time spent in the IZ or AZ, and the social interaction ratio (IR). IR was calculated as follows: (time in the IZ in the post-test)/(time in the AZ in the pre-test). Mice with IR < 1.0 were classified as susceptible, while the other mice were classified as resilient (Golden et al., 2011).

#### Sucrose preference test

Two 15-ml water bottles were placed in the cages for 24 h for habituation. Next, one water bottle was replaced with a 1% sucrose bottle. The amounts of water and 1% sucrose consumed over 12 h were measured. Sucrose preference was calculated as (sucrose consumption)/(total water and sucrose consumption) × 100 (Golden et al., 2011).

#### Locomotor activity test

Test mice were placed at the center of an open-field area (40 × 40 × 30 cm) and allowed to explore freely. Locomotor activity was measured as “counts” passing a set of infrared beams in the SCANET MV-40 (MELQUEST) for 60 min (Ma et al., 2017).

#### Tail suspension test

The tested mice were suspended from a suspension bar (12 cm in height) with their tails using adhesive tape for 6 min. Their movements were monitored, and immobility time was measured from 1–6 min (Machado et al., 2008).

#### Forced swimming test

The tested mice were placed in a transparent cylinder (21 cm in diameter × 22.5 cm in height) filled with water (23 ± 1°C, 18 cm in depth), and forced to swim for 6 min. Their movements were recorded using a SCANET MV-40 (MELQUEST), and the immobility time was measured from 1 to 6 min (K. Zhang et al., 2018).

### Microinfusions

After mice were anesthetized [medetomidine (0.3 mg/kg), midazolam (4.0 mg/kg), and butorphanol (5.0 mg/kg)], a guide cannula (AG-4, Eicom) was implanted into the skull with stainless steel screw and dental acrylic cement into the bilateral dSTR (AP, 0.5 mm; ML, ±2.0 mm; DV, 3.0 mm) or dRN (AP, −4.4 mm; ML, 0 mm; DV, 3.5 mm) using stereotaxic frame. Deschloroclozapine (DCZ; 0.1 μm/0.2 μl per side; Med Chem Express) or CGP36216 (GABA_(B)_ antagonist; 3 mm/0.1 μl per side; APExBIO) were infused into dSTR or dRN, respectively, by inserting the broken dialysis probe (A-I-4-01, Eicom) through the guide cannula using an injector EPS-64 microsyringe pump (Eicom). The effect of DCZ as a selective chemogenetic ligand for designer receptors (hM3Dq) has been previously reported (Nagai et al., 2020). The dose and timing of drug administration were based on previous studies (Nagai et al., 2020; Li et al., 2021). Ringer’s solution was used as the control.

### *In vivo* microdialysis

#### 5-HT measurement

5-HT measurements using *in vivo* microdialysis were performed as previously described (Miyamoto et al., 2017). The guide cannula was implanted with stainless steel screw and dental acrylic cement into the mice dSTR (AP, 0.5 mm; ML, ±2.0 mm; DV, 2.5 mm) using a stereotaxic frame. Twenty-four hours after surgery, Ringer’s solution was perfused (flow rate: 0.5 μl/min), and the dialysate was collected in a 12 min fraction through a dialysis probe (Eicom). The collected dialysate was injected into a high-performance liquid chromatography (HPLC) system (HTEC-500; Eicom). Two hours after probe insertion, baseline 5-HT levels were calculated as the average of four consecutive fractions (the difference between each value was <10%). DCZ was microinfused when the 5-HT baseline content was stable.

#### GABA measurement

GABA levels were measured using *in vivo* microdialysis as previously reported (Fu et al., 2016). The guide cannula was implanted into the mice dRN (AP, −4.4 mm; ML, 0 mm; DV, 3.0 mm), similar to the 5-HT measurement. Twenty-four hours after surgery, a dialysis probe (FX-I-4-01, 1-mm membrane length, Eicom) was inserted into the guide cannula, and the Ringer’s solution was perfused (flow rate: 0.5 μl/min). The dialysate was collected in a 30 min fraction using a dialysis probe. The collected dialysate was injected into an HPLC system (HTEC-500; Eicom). The average of four consecutive dialysates collected 4 h after probe insertion was defined as the baseline GABA level.

### Statistical analysis

All statistical analyses were performed using GraphPad Prism version 7. For comparing the results between the two groups, the Student’s *t* test was used. One-way ANOVA followed by Bonferroni’s *post hoc* test were used to compare the results of single-factor experiments between more than two groups. Two-way ANOVA followed by Tukey–Kramer’s or Bonferroni’s *post hoc* test, was used to compare the results of the double-factor experiments. Correlations were measured using Pearson’s correlation coefficient (*r*). All data are expressed as the mean ± SEM.

## Result

### 5-HT content in the dorsal striatum and sensitivity to RSDS

**Figure 1.**
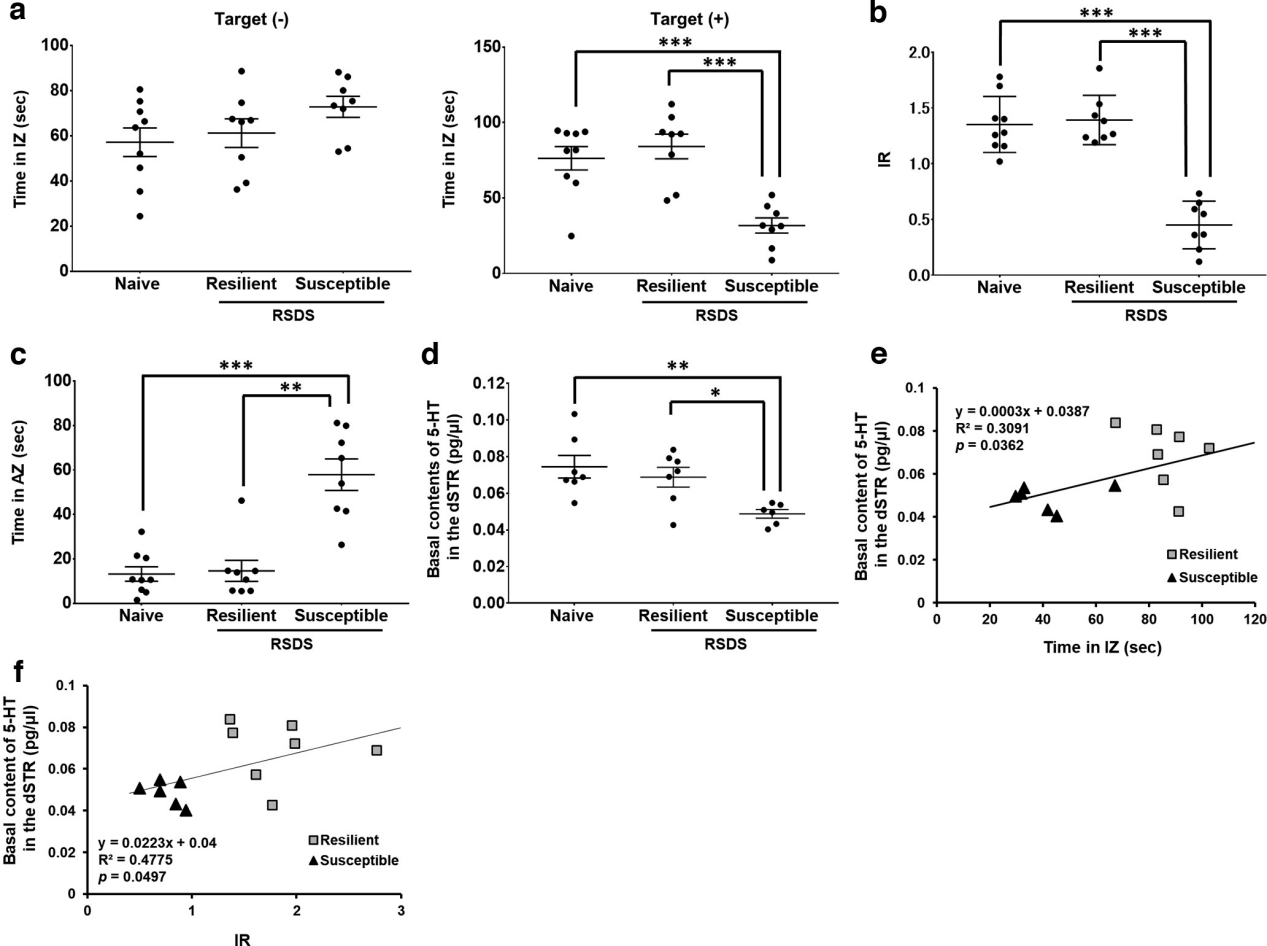
Decreased 5-HT content in the dSTR is correlated with depression-like behaviors. ***a–c***, Results of social interaction test. ***a***, Time in the interaction zone. Naive, *n* = 9; Resilient, *n* = 8; Susceptible, *n* = 8; ***p <* *0.005 versus susceptible mice (one-way ANOVA with Bonferroni’s *post hoc* tests). ***b***, Interaction ratio. Naive, *n* = 9; Resilient, *n* = 8; Susceptible, *n* = 8; ***p <* *0.005 versus susceptible mice (one-way ANOVA with Bonferroni’s *post hoc* tests). ***c***, Time in avoidance zone. Naive, *n* = 9; Resilient, *n* = 8; Susceptible, *n* = 8; ***p <* *0.005 versus susceptible mice, **p <* *0.01 versus susceptible mice (one-way ANOVA with Bonferroni’s *post hoc* tests). ***d***, Quantitative of basal 5-HT levels in the dSTR. Naive, *n* = 7; Resilient, *n* = 7; Susceptible, *n* = 6; **p <* *0.01 versus susceptible mice, *p <* *0.05 versus susceptible mice (one-way ANOVA with Bonferroni’s *post hoc* tests). ***e, f***, Correlation of basal 5-HT content in the dSTR with the social interaction ability assessed by the time spent in interaction zone (***e***) and by interaction ratio (***f***) after RSDS. Resilient, *n* = 7; Susceptible, *n* = 6 (Pearson’s correlation test).

Depression-like behaviors were assessed in mice after exposure to RSDS using a social interaction test. No differences in the time spent in the IZ were observed among the three groups during the pretest (Fig. 1*a*, left). However, the SI time decreased in susceptible mice compared with stress-naive and resilient mice during the post-test (Fig. 1*a*, right; *F*_(2,22)_ = 15.06, *p *<* *0.0001; one-way ANOVA). The IR was also significantly lower in susceptible mice than in stress-naive and stress-resilient mice (Fig. 1*b*; *F*_(2,22)_ = 43.54, *p *<* *0.0001; one-way ANOVA). In contrast, the time spent in the AZ was significantly increased in susceptible mice compared with that in stress-naive and resilient mice (Fig. 1*c*; *F*_(2,22)_ = 24.10, *p *<* *0.0001; one-way ANOVA). These results indicated that the RSDS was correctly performed, allowing us to obtain stress-resilient or stress-susceptible groups of animals.

We have previously demonstrated the relationship between 5-HT in the dSTR and Shati/Nat8l-induced stress vulnerability to RSDS (Uno et al., 2019). The basal 5-HT content in the dSTR of C57BL/6J mice exposed to RSDS was measured using *in vivo* microdialysis after social interaction tests. They were significantly decreased in susceptible, but not resilient, mice compared with those in stress-naive mice (Fig. 1*d*; *F*_(2,17)_ = 6.55, *p *=* *0.0078; one-way ANOVA), suggesting the involvement of dSTR 5-HT in stress sensitivity underlying the pathogenesis of depression. These results are consistent with those of a previous study, in which a reduction in 5-HT content was observed in mice with dSTR-specific Shati/Nat8l overexpression (referred to as dSTR-Shati OE; Miyamoto et al., 2017).

### Activation of serotonergic neurons from the dorsal raphe nucleus using the designer receptors exclusively activated by designer drugs (DREADD) system and 5-HT levels in the dorsal striatum

**Figure 2.**
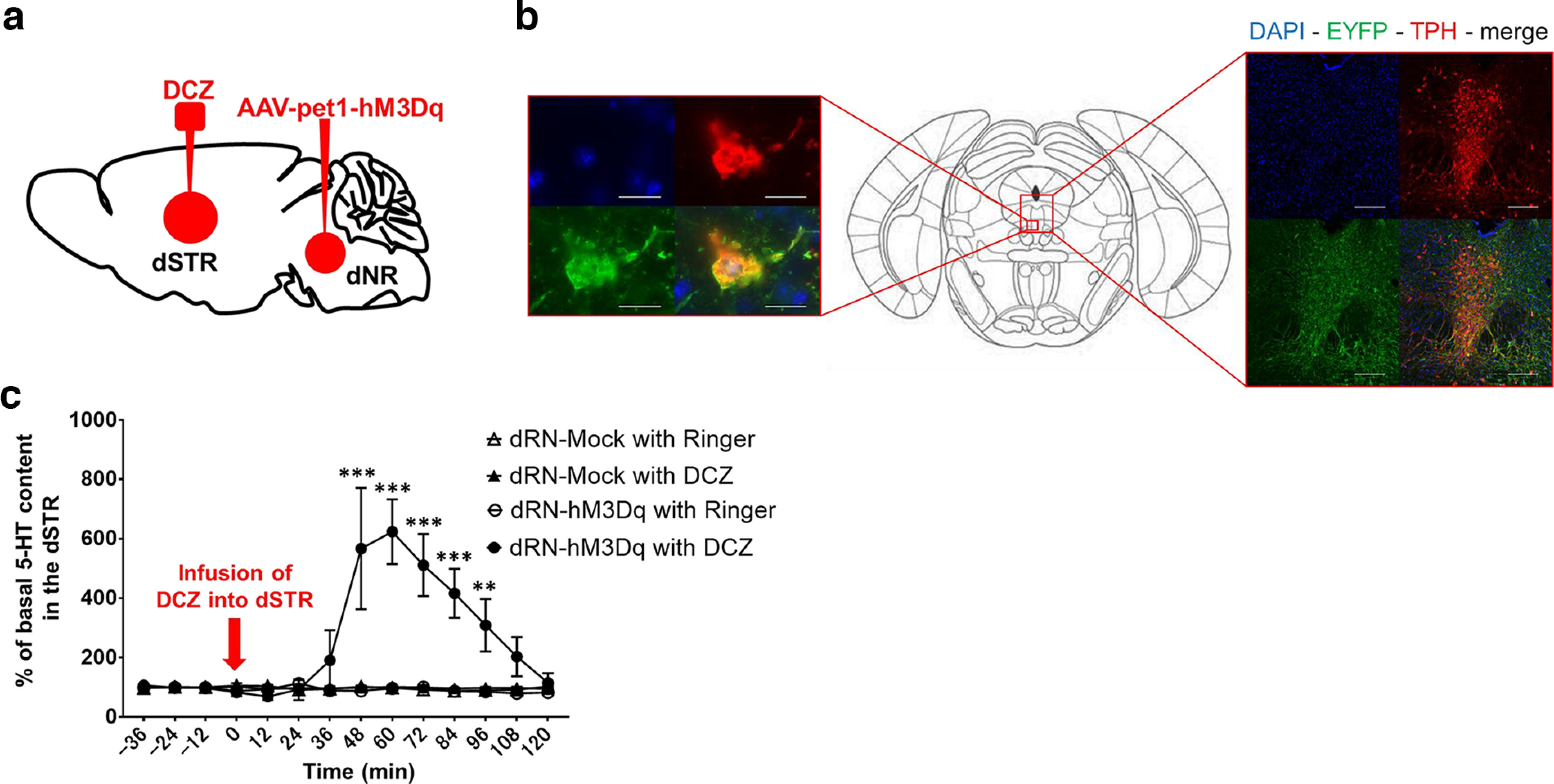
Pharmacogenetic activation of serotonergic neurons in the dRN. ***a***, Schematic of microinjection of AAV vectors into the dRN and microinfusion of DCZ into the dSTR. ***b***, Representative images of injection sites (dRN). TPH-positive cells: red signal; EYFP-positive cells: green signal. Entire portion (left), Scale bars: 200 μm. Magnified portion (right), Scale bars: 20 μm. ***c***, 5-HT content in dSTR collected every 12 min for 2 h from the time DCZ was microinfused. dRN-Mock with Ringer, *n* = 4; dRN-Mock with DCZ, *n* = 5; dRN-hM3Dq with Ringer, *n* = 5; dRN- hM3Dq with DCZ, *n* = 5; ***p <* *0.005 versus dRN-Mock with Ringer, **p <* *0.05 versus dRN-Mock with Ringer (two-way ANOVA with Bonferroni’s *post hoc* tests).

The dRN contains major serotonergic populations that project to numerous areas of the brain including the dSTR (Azmitia and Segal, 1978; Pollak et al., 2014), thus, we focused on studying the serotonergic system in dRN. The AAV-pet1-hM3Dq vector (Hasegawa et al., 2014) was microinjected into the dRN, and deschloroclozapine (DCZ) was locally administered to effectively activate serotonergic neurons from the dRN expressing hM3Dq (Fig. 2*a*). The expression of EYFP (green signals) was detected in serotonergic neurons (TPH-positive cells; red signals) in the dRN (Fig. 2*b*). We also microinjected the AAV-pet1-Mock vector into the dRN of mice in the control group. Forty-eight minutes after DCZ microinfusion, a significant increase in dSTR 5-HT content was observed for 1 h in dRN-hM3Dq mice treated with DCZ, but not in the groups injected with the AAV-pet1-Mock vector or treated Ringer’s solution (Fig. 2*c*; interaction effect: *F*_(39,201)_ = 5.268, *p *<* *0.0001, two-way ANOVA).

### Striatal Shati/Nat8l expression and vulnerability to stress

**Figure 3.**
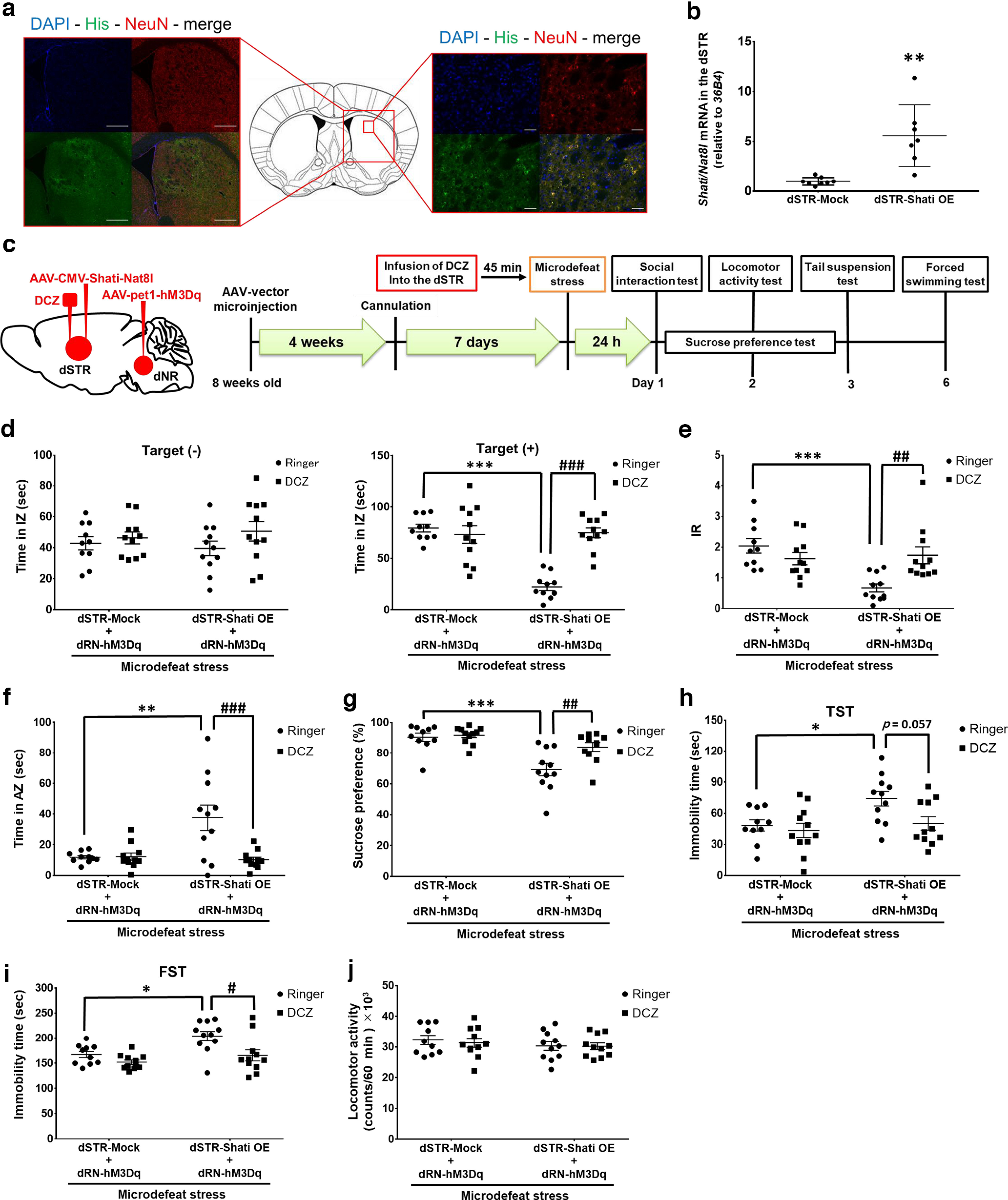
Upregulation of 5-HT content in the dSTR induced resilience to stress in dSTR-Shati OE mice. ***a***, Representative images of injection sites (dSTR). Shati/Nat8l (His-positive cells): green signal, NeuN-positive cells: red signal. Entire portion (right), Scale bars: 500 μm. Magnified portion (left), Scale bars: 50 μm. ***b***, Relative expression level of *Shati/Nat8l* mRNA in the dSTR. dSTR-Mock, *n* = 8; dSTR-Shati OE, *n* = 7; **p <* *0.01 versus dSTR-Mock mice (student *t* test). ***c***, Schematic of microinjection and microinfusion and the timeline of experiments. ***d–f***, Results of social interaction test. ***d***, Time in the interaction zone. dSTR-Mock with ringer, *n* = 10; dSTR-Mock with DCZ, *n* = 11; dSTR-Shati OE with Ringer, *n* = 11; dSTR-Shati OE with DCZ, *n* = 11; ***p <* *0.005 versus dSTR-Mock with Ringer; ###*p *<* *0.005 versus dSTR-Shati OE with DCZ (two-way ANOVA with Bonferroni’s *post hoc* tests). ***e***, Interaction ratio. dSTR-Mock with Ringer, *n* = 10; dSTR-Mock with DCZ, *n* = 11; dSTR-Shati OE with Ringer, *n* = 11; dSTR-Shati OE with DCZ, *n* = 11; ***p <* *0.005 versus dSTR-Mock with Ringer; ##*p *<* *0.01 versus dSTR-Shati OE with DCZ (two-way ANOVA with Bonferroni’s *post hoc* tests). ***f***, Time in avoidance zone. dSTR-Mock with Ringer, *n* = 10; dSTR-Mock with DCZ, *n* = 11; dSTR-Shati OE with Ringer, *n* = 11; dSTR-Shati OE with DCZ, *n* = 11; **p < 0.01 versus dSTR-Mock with Ringer; ###*p* < 0.005 versus dSTR-Shati OE with DCZ (two-way ANOVA with Bonferroni’s *post hoc* tests). ***g***, Result of sucrose preference test. dSTR-Mock with Ringer, *n* = 10; dSTR-Mock with DCZ, *n* = 11; dSTR-Shati OE with Ringer, *n* = 11; dSTR-Shati OE with DCZ, *n* = 11; ***p <* *0.005 versus dSTR-Mock with ringer; ##*p *<* *0.01 versus dSTR-Shati OE with DCZ (two-way ANOVA with Bonferroni’s *post hoc* tests). ***h***, Immobility time in tail suspension test. dSTR-Mock with Ringer, *n* = 10; dSTR-Mock with DCZ, *n* = 11; dSTR-Shati OE with Ringer, *n* = 11; dSTR-Shati OE with DCZ, *n* = 11; *p <* *0.05 versus dSTR-Mock with Ringer (two-way ANOVA with Tukey’s *post hoc* tests). ***i***, Immobility time in a forced swimming test. dSTR-Mock with Ringer, *n* = 10; dSTR-Mock with DCZ, *n* = 11; dSTR-Shati OE with Ringer, *n* = 11; dSTR-Shati OE with DCZ, *n* = 11; *p <* *0.05 versus dSTR-Mock with Ringer; #*p *<* *0.05 versus dSTR-Shati OE with DCZ (two-way ANOVA with Bonferroni’s *post hoc* tests). ***j***, Results of locomotor activity test. dSTR-Mock with Ringer, *n* = 10; dSTR-Mock with DCZ, *n* = 11; dSTR-Shati OE with Ringer, *n* = 11; dSTR-Shati OE with DCZ, *n* = 11 (two-way ANOVA with Bonferroni’s *post hoc* tests).

Furthermore, we investigated the contribution of serotonergic neurons projecting from the dRN to the dSTR to stress sensitivity. As mentioned above, we demonstrated that dSTR Shati/Nat8l regulates stress sensitivity and that dSTR-Shati OE mice are susceptible to stress (Uno et al., 2019). The dSTR-Shati OE mice were obtained by microinjecting the AAV-CMV-Shati/Nat8l vector into their dSTR. The AAV-CMV-Mock vector was injected into the dSTR of control mice (referred to as dSTR-Mock). As shown in Figure 3*a*, His-positive cells (green signals) were detected in the dSTR of the dSTR-Shati OE mice. We confirmed the overexpression of *Shati/Nat8l* mRNA in dSTR using real-time RT-PCR (Fig. 3*b*; *t*_(13)_ = 4.199, *p *=* *0.0010; Student’s *t* test). These mice were exposed to microdefeat stress (subthreshold social stress) to assess stress sensitivity, and depression-like behaviors were assessed following the protocol (Fig. 3*c*). Bilateral microinjection of AAV-CMV-Shati/Nat8l or Mock vectors into the mouse dSTR and the AAV-pet1-hM3Dq vector into the dRN was performed. DCZ was microinfused 45 min before microdefeat stress to adjust the timing during the upregulation of 5-HT content in the dSTR using a guide cannula bilaterally implanted. Twenty-four hours after microdefeat stress, behavioral tests were performed to assess the depression-like behaviors induced by RSDS exposure. There was no difference in the SI time between all groups in the pre-test (Fig. 3*d*, left). No difference in SI time after microdefeat stress was observed between dSTR-Mock mice treated with Ringer’s solution or DCZ (Fig. 3*d*, right), suggesting that DCZ had no effect on social interaction in dSTR-Mock mice. dSTR-Shati OE mice treated with Ringer’s solution showed a significant decrease in social interactions compared with dSTR-Mock mice. Activation of dRN-dSTR serotonergic neurons in dSTR-Shati OE mice by DCZ treatment prevented this decrease (Fig. 3*d*, right; interaction effect: *F*_(1,39)_ = 27.26, *p *<* *0.0001; two-way ANOVA). Furthermore, dSTR-Shati OE mice treated with Ringer’s solution, but not those treated with DCZ, showed lower IR after microdefeat stress (Fig. 3*e*; interaction effect: *F*_(1,39)_ = 11.71, *p *=* *0.0015; two-way ANOVA). Notably, the proportion of the stress-resilient group after microdefeat stress exposure was strongly elevated by DCZ treatment in dSTR-Shati cells. Although 27.2% of dSTR-Shati OE mice treated with Ringer’s solution showed stress resilience after exposure to microdefeat stress (*n* = resilient/susceptible: 3/8), all dSTR-Shati OE mice treated with DCZ belonged to the stress-resilient group (*n* = resilient/susceptible: 11/0). The decreased AZ time in dSTR-Shati OE mice treated with Ringer’s solution was prevented by microinfusion of DCZ into the dSTR (Fig. 3*f*; interaction effect: *F*_(1,39)_ = 9.390, *p *=* *0.0039; two-way ANOVA). In the sucrose preference test, microdefeat stress decreased sucrose preference in ringer-treated dSTR-Shati OE mice, but not in DCZ-treated dSTR-Shati OE mice (Fig. 3*g*; interaction effect: *F*_(1,39)_ = 5.059, *p *=* *0.0302; two-way ANOVA). In the tail suspension test (Fig. 3*h*; interaction effect: *F*_(1,39)_ = 2.152, *p *=* *0.1504; two-way ANOVA), No increase in the immobility time assessed in the FST was observed in DCZ-treated dSTR-Shati OE mice compared with ringer-treated dSTR-Shati OE mice (Fig. 3*i*; interaction effect: *F*_(1,39)_ = 1.961, *p *=* *0.1693; two-way ANOVA). To exclude the possibility that our results were affected by experimental methods, such as microinjection or microinfusion into the dSTR and dRN, a locomotor activity test was performed to assess basic motor function. We confirmed that these procedures had no influence on motor function (Fig. 3*j*). These results indicate that overexpression of Shati/Nat8l in the dSTR induces vulnerability to social stress, possibly by controlling striatal 5-HT levels through serotonergic neurons projected from the dRN to the dSTR.

### dRN GABA content in stress susceptible mice and dSTR-Shati OE mice and correlation with the social interaction behavior

**Figure 4.**
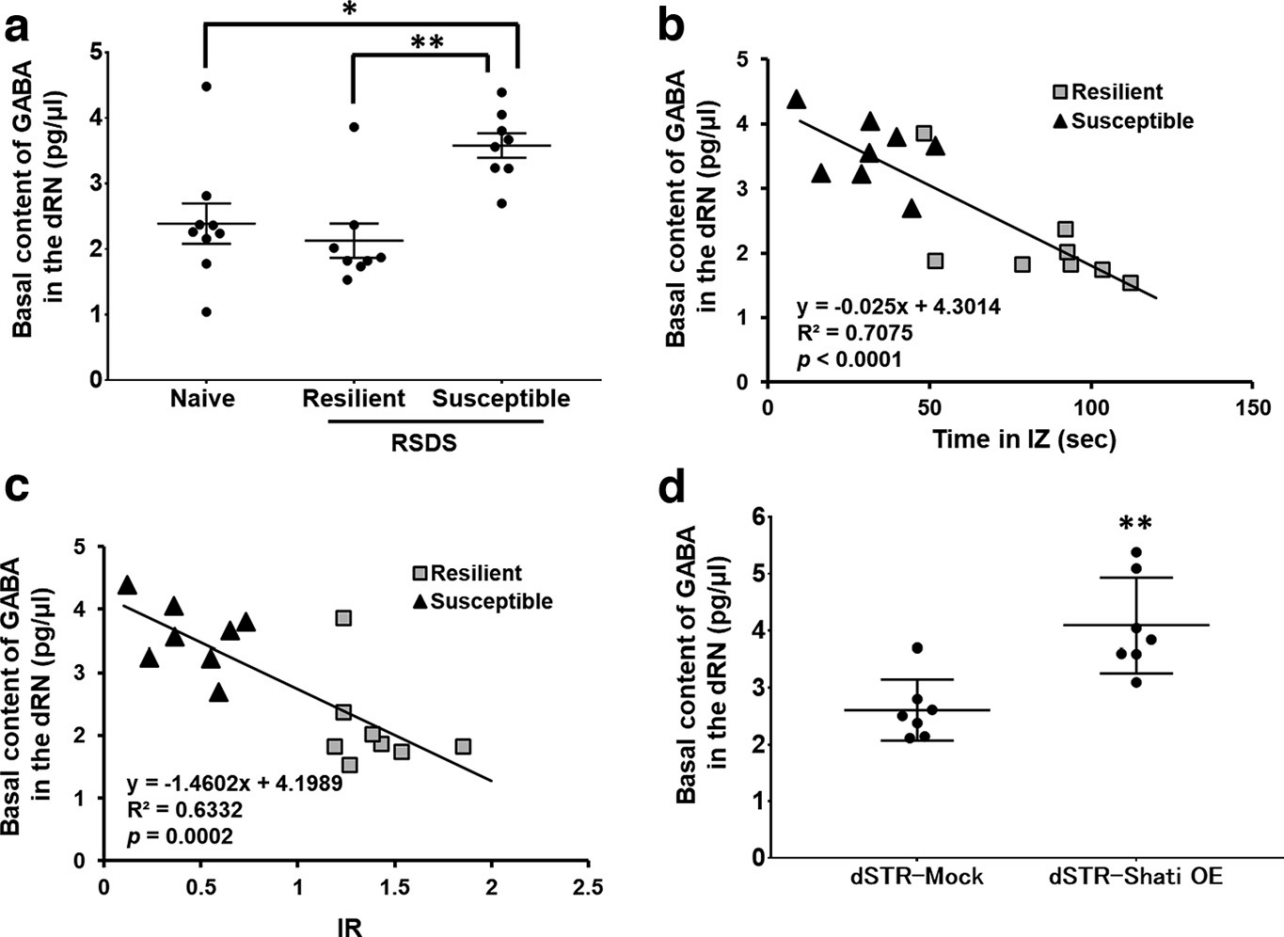
GABA content in the dRN is correlated with depression-like behaviors and controlled by Shati/Nat8l in the dSTR. ***a***, Quantitative basal GABA content in the dRN. Naive, *n* = 9; Resilient, *n* = 8; Susceptible, *n* = 8; **p <* *0.01 versus susceptible mice; *p <* *0.05 versus susceptible mice (one-way ANOVA with Bonferroni’s *post hoc* tests). ***b***, ***c***, Correlation of basal GABA content in the dRN with the social interaction ability assessed by the time spent in interaction zone (***b***) and by interaction ratio (***c***) after RSDS. Resilient, *n* = 8; Susceptible, *n* = 8 (Pearson’s correlation test). ***d***, Quantitative basal GABA content in the dRN. Mock, *n* = 8; dSTR-Shati, *n* = 7; **p <* *0.01 versus Mock mice (student *t* test).

Next, we investigated the regulatory mechanisms of the dSTR Shati/Nat8l on the activity of serotonergic neurons projecting from the dRN to the dSTR. In our previous study, we demonstrated the existence of neuronal projections from the dSTR to the dRN (Uno et al., 2019). Medium-sized spiny neurons (MSNs) are the most abundant cells in dSTR, accounting for 95% of all neurons (Graveland and DiFiglia, 1985; Kreitzer, 2009). Given that MSNs are GABAergic cells and major projection neurons in the dSTR (Mao et al., 2019), we investigated the involvement of GABAergic neurons in the regulation of the dRN serotonergic system. We found that after exposure to RSDS, stress-susceptible mice exhibited significantly higher basal GABA concentrations than in naive mice. This difference was not present between stress-resilient and naive mice (Fig. 4*a*; *F*_(2,22)_ = 8.423, *p *=* *0.0019; one-way ANOVA). Furthermore, basal dRN GABA content was negatively correlated with indicators of social interaction ability, including IZ time and IR (Fig. 4*b*,*c*; vs time in the IZ: *r* = −0.841, *p *<* *0.0001; vs social IR: *r* = −0.796, *p *=* *0.0002; Pearson’s correlation test). This suggests the potential involvement of dRN-GABA in the pathogenesis of depression.

We investigated the relationship between dSTR Shati/Nat8l expression and dRN GABA content. dSTR-Shati OE mice exhibited vulnerability to social defeat stress and showed an increase in basal dRN GABA content compared with dSTR-Mock mice (*t*_(12)_ = 3.939, *p *=* *0.0020; Student’s *t* test; Fig. 4*d*). These results suggest that the dSTR Shati/Nat8l regulates stress sensitivity via GABAergic neurons.

### The function of GABA in the dorsal raphe nucleus on the stress sensitivity

**Figure 5.**
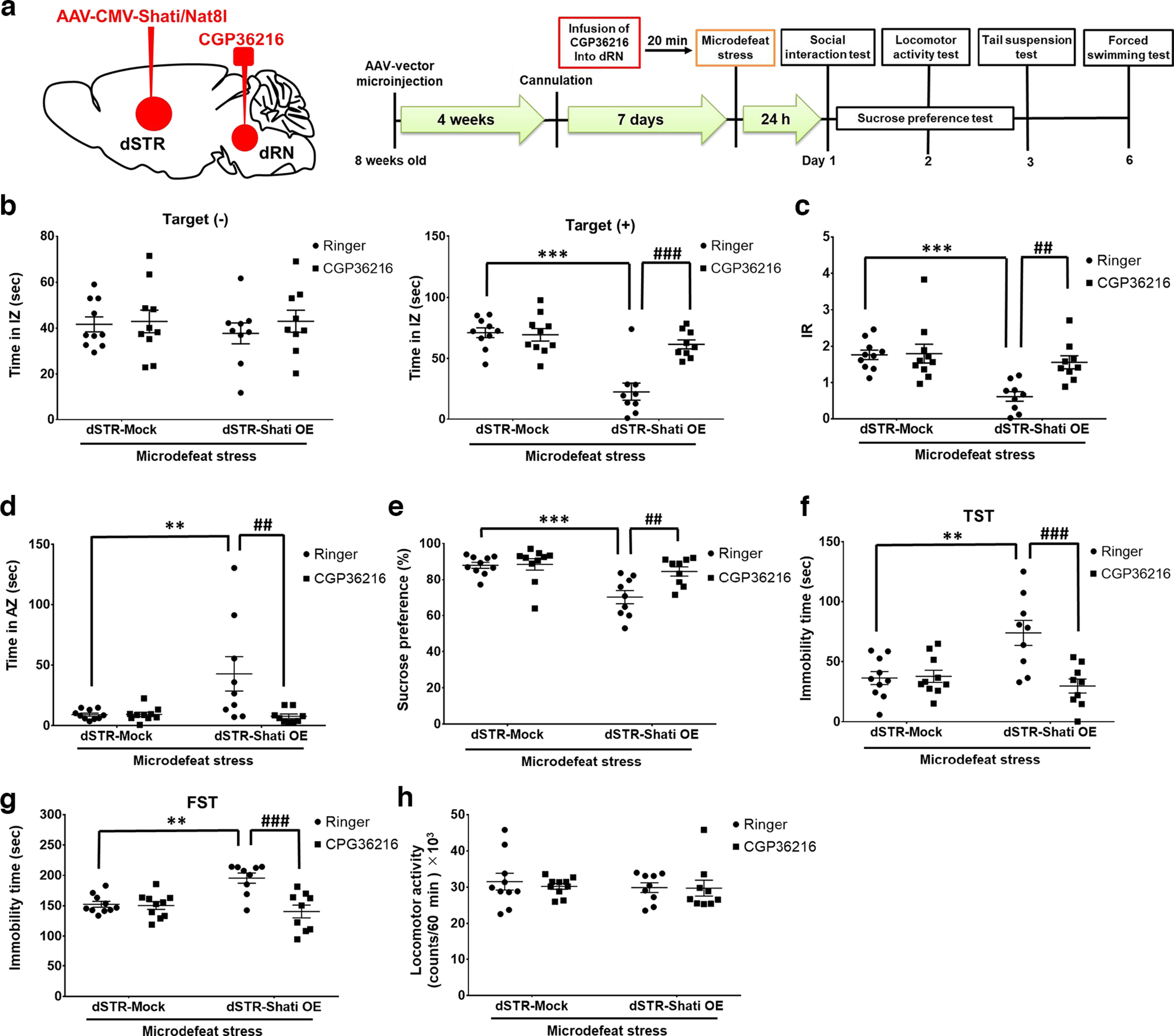
Blockade of GABA signaling in the dRN suppressed stress vulnerability in dSTR-Shati OE mice. ***a***, Schematic of microinjection and microinfusion and the timeline of experiments. ***b–d***, Results of social interaction test. ***b***, Time in the interaction zone. dSTR-Mock with ringer, *n* = 10; dSTR-Mock with CGP36216, *n* = 11; dSTR-Shati OE with Ringer, *n* = 9; dSTR-Shati OE with CGP36216, *n* = 9; ***p <* *0.005 versus dSTR-Mock with Ringer; ###*p *<* *0.005 versus dSTR-Shati OE with CGP362160 (two-way ANOVA with Bonferroni’s *post hoc* tests). ***c***, Interaction ratio. dSTR-Mock with Ringer, *n* = 10; dSTR-Mock with CGP36216, *n* = 10; dSTR-Shati OE with Ringer, *n* = 9; dSTR-Shati OE with CGP36216, *n* = 9; ***p <* *0.005 versus dSTR-Mock with Ringer; ##*p *<* *0.01 versus dSTR-Shati OE with CGP36216 (two-way ANOVA with Bonferroni’s *post hoc* tests). ***d***, Time in avoidance zone. dSTR-Mock with Ringer, *n* = 10; dSTR-Mock with CGP36216, *n* = 10; dSTR-Shati OE with Ringer, *n* = 9; dSTR-Shati OE with CGP36216, *n* = 9; **p <* *0.01 versus dSTR-Mock with Ringer; ##*p *<* *0.01 versus dSTR-Shati OE with CGP36216 (two-way ANOVA with Bonferroni’s *post hoc* tests). ***e***, Result of sucrose preference test. dSTR-Mock with Ringer, *n* = 10; dSTR-Mock with CGP36216, *n* = 10; dSTR-Shati OE with Ringer, *n* = 9; dSTR-Shati OE with CGP36216, *n* = 9; ***p <* *0.005 versus dSTR-Mock with ringer; ##*p *<* *0.01 versus dSTR-Shati OE with CGP36216 (two-way ANOVA with Bonferroni’s *post hoc* tests). ***f***, Immobility time in tail suspension test. dSTR-Mock with Ringer, *n* = 10; dSTR-Mock with CGP36216, *n* = 10; dSTR-Shati OE with Ringer, *n* = 9; dSTR-Shati OE with CGP36216, *n* = 9; **p <* *0.01 versus dSTR-Mock with Ringer; ###*p *<* *0.005 versus dSTR-Shati OE with CGP36216 (two-way ANOVA with Tukey’s *post hoc* tests). ***g***, Immobility time in a forced swimming test. dSTR-Mock with Ringer, *n* = 10; dSTR-Mock with CGP36216, *n* = 10; dSTR-Shati OE with Ringer, *n* = 9; dSTR-Shati OE with CGP36216, *n* = 9; **p <* *0.01 versus dSTR-Mock with Ringer; ###*p *<* *0.005 versus dSTR-Shati OE with CGP36216 (two-way ANOVA with Tukey’s *post hoc* tests). ***h***, Results of locomotor activity test. dSTR-Mock with Ringer, *n* = 10; dSTR-Mock with CGP36216, *n* = 10; dSTR-Shati OE with Ringer, *n* = 9; dSTR-Shati OE with CGP36216, *n* = 9 (two-way ANOVA with Bonferroni’s *post hoc* tests).

Next, we assessed the role of dRN GABA in stress sensitivity. We microinfused CGP36216, a GABA_(B)_ receptor antagonist, into the dRN of dSTR-Shati OE mice before microdefeat stress and depressive behaviors were investigated following the schedule (Fig. 5*a*). The IZ time between all groups in the pretest did not change (Fig. 5*b*, left). Although there was a difference in IZ time after microdefeat stress between dSTR-Mock mice and dSTR-Shati OE mice treated with Ringer’s solution, inhibition of GABA_(B)_ signaling in the dRN by CGP36216 suppressed the decrease in IZ time observed in dSTR-Shati OE mice in the post-test (Fig. 5*b*, right; interaction effect: *F*_(1,34)_ = 15.66, *p *=* *0.0004; two-way ANOVA). The decrease in IR and increase in AZ time after microdefeat stress exposure in dSTR-Shati OE mice also disappeared in dSTR-Shati OE mice treated with CGP36216 (Fig. 5*c*,*d*; IR: interaction effect: *F*_(1,34)_ = 5.973, *p *=* *0.0199; AZ time: interaction effect: *F*_(1,34)_ = 6.545, *p *=* *0.0151; two-way ANOVA). Although sucrose preference in ringer-treated dSTR-Shati OE mice decreased after microdefeat stress, CGP36216-treated dSTR-Shati OE mice did not show this behavior (Fig. 5*e*; interaction effect: *F*_(1,34)_ = 6.080, *p *=* *0.0189; two-way ANOVA). Furthermore, dSTR-Shati OE mice treated with Ringer’s solution, but not those treated with CGP36216, showed longer immobility time after microdefeat stress exposure in the tail suspension and forced swimming tests (Fig. 5*f*,*g*; tail suspension test: interaction effect: *F*_(1,34)_ = 11.01, *p *=* *0.0022; forced swimming test: interaction effect: *F*_(1,34)_ = 11.96, *p *=* *0.0015; two-way ANOVA). Impaired motor function was not observed in the CGP36216-treated mice (Fig. 5*h*). These results indicate that dSTR-Shati/Nat8l-induced stress vulnerability is mediated by GABA_(B)_ signaling in the dRN and imply that inhibition of this signaling results in stress resistance.

**Figure 6.**
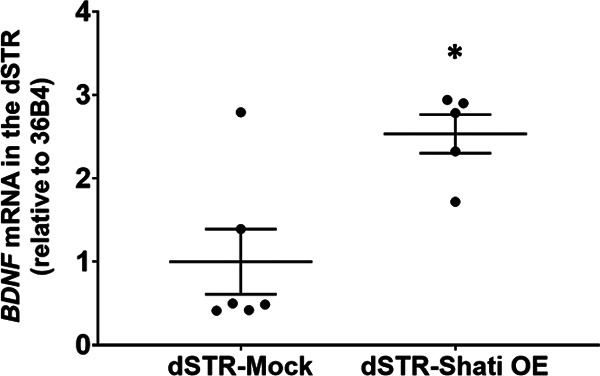
Overexpression of Shat/Nat8l enhanced BDNF expression in the dSTR. Relative expression level of *Shati/Nat8l* mRNA in the dSTR. dSTR-Mock, *n* = 6; dSTR-Shati OE, *n* = 5; *p <* *0.05 versus dSTR-Mock mice (student *t* test).

To clarify the regulatory mechanism of GABA neurotransmitter by Shati/Nat8l, we focused on brain-derived neurotrophic factor (BDNF). The role of BDNF in the regulation of GABAergic synapse plasticity were reported (Brady et al., 2018). Furthermore, we previously reported that knock-down of Shati/Nat8l decreased BDNF expression in the dSTR, and BDNF in the dSTR determined the stress sensitivity (Miyanishi et al., 2021). *BDNF* mRNA in dSTR increased in dSTR-Shati OE mice compared with dSTR-Mock mice (Fig. 6; *t*_(9)_ = 3.201, *p *=* *0.0108; Student’s *t* test).

## Discussion

Our study provides evidence that the neuronal circuitry between the dSTR and the dRN, which controls 5-HT levels, is essential for the regulation of stress sensitivity. Depression was accompanied by decreased dSTR 5-HT levels in stress-susceptible mice after RSDS exposure. Therefore, the dSTR 5-HT content interferes with social interaction behaviors. Similar changes in dSTR 5-HT levels were observed in dSTR-Shati OE mice, which were vulnerable to stress. Increased vulnerability to stress induced by the overexpression of Shati/Nat8l, was prevented by the specific activation of dRN-dSTR serotonergic neurons using the DREADD system. Thus, Shati/Nat8l regulates stress sensitivity by modulating 5-HT levels in the dSTR via dRN-dSTR serotonergic neurons. Additionally, increased dRN-GABA content was observed only in RSDS stress-susceptible mice. Shati OE mice showed similar changes in dRN GABA content. dRN GABA content is correlated with social interaction behavior and the inhibition of GABA signaling in dRN-induced stress resilience, suggesting that GABA levels in the dRN determine stress sensitivity. The present study suggests that there is an interactive neuronal network connecting the dSTR and dRN, which is controlled by Shati/Nat8l, and regulates stress sensitivity by influencing GABA release in the dRN and 5-HT release in the dSTR. Thus, our study reveals the underlying molecular mechanisms that contribute to stress susceptibility.

RSDS-induced depression-like behaviors, such as impaired social interaction ability, lack of pleasure (anhedonia), and helplessness in stressful situations (Carnevali et al., 2020), reflect the symptoms of depression in humans. These depression-like behaviors are prevented by the administration of SSRIs (Bourke et al., 2014; Liu et al., 2020). SSRI microinfusion into the dSTR prevented vulnerability to RSDS and even subthreshold social stress in dSTR-Shati OE mice (Uno et al., 2019), indicating that 5-HT levels in the dSTR play an important role in stress sensitivity. These results are consistent with those obtained in the present study.

The incidence of depression is higher in women than in men (Altemus et al., 2014). Although RSDS protocols for female mice have been established (Takahashi et al., 2017), male mice were used in this study because microdefeat stress requires reasonable aggressiveness of the used ICR mice, and it was difficult to apply microdefeat stress protocols to female mice in the present study. We also investigated the role of the dSTR-dRN neural circuitry in stress sensitivity without considering the possible influence of the estrous cycle. However, an experiment including female mice should be considered in future studies using appropriate protocols of social defeat stress or other female-specific social stress models, such as social crowding stress (Furman et al., 2022), because of the need to develop treatments for all patients with depression.

Although the ventral striatum (nucleus accumbens) is involved in the pathogenesis of stress or depression (Nestler and Carlezon, 2006; Heshmati et al., 2020), the involvement of the dSTR in these pathologies was not demonstrated until our previous study (Miyamoto et al., 2017). The dSTR contributes to negative decision-making and emotional behavior (Amemori et al., 2018; Klawonn et al., 2021). Among the neuronal networks with other brain regions that modulate such functions (Lago et al., 2017; Cox and Witten, 2019), we considered the involvement of bidirectional dSTR-dRN neuronal interplay in the present study. Given that 5-HT in the dSTR plays an important role in stress sensitivity, we focused on the dRN serotonergic system. Most dRN neurons are serotonergic and project to the entire brain (Ishimura et al., 1988; Pollak et al., 2014). We demonstrated that dRN-dSTR serotonergic neurons are downstream of striatal Shati/Nat8l and that their activation induces stress resilience. To clarify the mechanisms underlying the regulation of dRN-dSTR serotonergic neurons by dSTR Shati/Nat8l, we hypothesized that dSTR-dRN GABAergic neurons were present. Medium-sized spiny neurons (MSNs) are GABAergic cells, which are the major projection neurons of the striatum (Mao et al., 2019), and are the most abundant cell type. ∼95% of the neurons in the striatum are MSN cells (Kemp and Powell, 1971; Babenko et al., 2020). Here, we demonstrated that elevated dRN GABA content was observed in stress-susceptible but not in resilient mice subjected to RSDS. Moreover, dRN GABA levels were strongly correlated with social interaction behavior indicators. These results are consistent with our other findings that dSTR-Shati OE mice exhibit increased vulnerability to social stress and show upregulation of dRN GABA content. We also demonstrated that blocking of GABA signaling in the dRN inhibited stress susceptibility in dSTR-Shati OE mice, suggesting that dRN GABA induces stress vulnerability through suppression of serotonergic neurons projecting from the dRN to the dSTR because GABA functions as an inhibitory neurotransmitter (Petroff, 2002). Decreased dSTR 5-HT content in dSTR-Shati OE mice and stress-susceptible mice after RSDS is in accordance with the fact that dRN GABA content in these mice is increased. Previous reports showed that the antidepressant effect of the potentiation of 5-HT neurons is mediated by decreased GABA signaling in the dRN (Asaoka et al., 2017), further supporting our suggestion that GABA neurotransmission suppress serotonergic neuron in the dRN. GABA_(B)_ receptor antagonists were used in this study because previous studies have shown that blockage of GABA_(B)_ receptors induces an antidepressant effect (Mombereau et al., 2004; Alexander, 2017) and activation of GABA_(B)_ receptors in the dRN attenuates 5-HT neuronal activity (Tao and Auerbach, 2000), indicating the depressant effect of GABA_(B)_ signaling in the dRN.

Although we emphasized the role of dSTR 5-HT in the present study, our results do not deny the possible involvement of 5-HT from other regions in the pathology of depression. In fact, 5-HT imbalances in the hippocampus and medial prefrontal cortex (mPFC) contribute to this imbalance (Le et al., 2016; Belleau et al., 2019). Depression-like behavior in rodents is prevented by the microinfusion of 5-HT or a 5-HT receptor agonist into the hippocampus or mPFC (Luo et al., 2008; Fukumoto et al., 2018). However, the roles of 5-HT in various regions may differ, and the dSTR 5-HT determines stress sensitivity. The dSTR-Shati OE mice, which had reduced STR 5-HT levels, did not exhibit depression-like behaviors without social defeat stress. Furthermore, activation of dSTR-dRN serotonergic neurons in mock mice did not induce antidepressant behaviors, indicating that striatal 5-HT deficits impair stress sensitivity but not depression-like behaviors. TPH-2 knock-in mice, which show a decrease in brain 5-HT by ∼70%, do not show depression-like behavior without social defeat stress either (Sachs et al., 2015). These reports support our hypothesis on the role of 5-HT in stress sensitivity.

Shati/Nat8l regulates neural circuitry between the dSTR and dRN. One possible regulatory mechanism is BDNF. dSTR Shati/Nat8l knock-down mice exhibit reduced *BDNF* mRNA and protein levels in the dSTR and resilience to high-intensity RSDS exposure (Miyanishi and Nitta, 2021). Furthermore, inhibition of BDNF signaling by ANA-12 (a tyrosine protein kinase inhibitor) induces stress resilience (Miyanishi et al., 2021). These results suggest that the BDNF levels in the dSTR are regulated by Shati/Nat8l and are involved in stress sensitivity. Upregulation of *BDNF* mRNA levels was observed in stress-susceptible but not resilient mice subjected to RSDS (Miyanishi et al., 2021), and we confirmed that dSTR Shati/Nat8l OE mice have higher *BDNF* mRNA levels in the dSTR compared with those in mock mice. It is possible that the BDNF increase in the dSTR induced by Shati/Nat8l overexpression could account for the high susceptibility to stress in dSTR-Shati OE mice. BDNF promotes neurogenesis, neuronal excitability, and plasticity by exerting neurotrophic functions (Ferrini and De Koninck, 2013; Colucci-D’Amato et al., 2020; Yang et al., 2020). In the present study, we observed an increase in dRN-GABA content in stress-susceptible and dSTR-Shati OE mice. The activation of GABAergic neurons projecting from the dSTR to the dRN may be induced by the upregulation of striatal BDNF levels.

While conventional antidepressants do not have efficacy in 30% of patients with treatment resistance, ketamine induces rapid effects even in those patients (Rosenblat et al., 2019). The activation of the serotoninergic system in the dRN has been reported as a therapeutic mechanism of ketamine (Fukumoto et al., 2016; Chaki and Fukumoto, 2019). Targeting dSTR Shati/Nat8l might result in effects similar to those of ketamine through direct modulation of the dRN serotonergic system. dSTR Shati/Nat8l might be a promising candidate for developing novel antidepressant therapies that are useful for all patients, including those with refractory forms of the disease.

**Figure 7.**
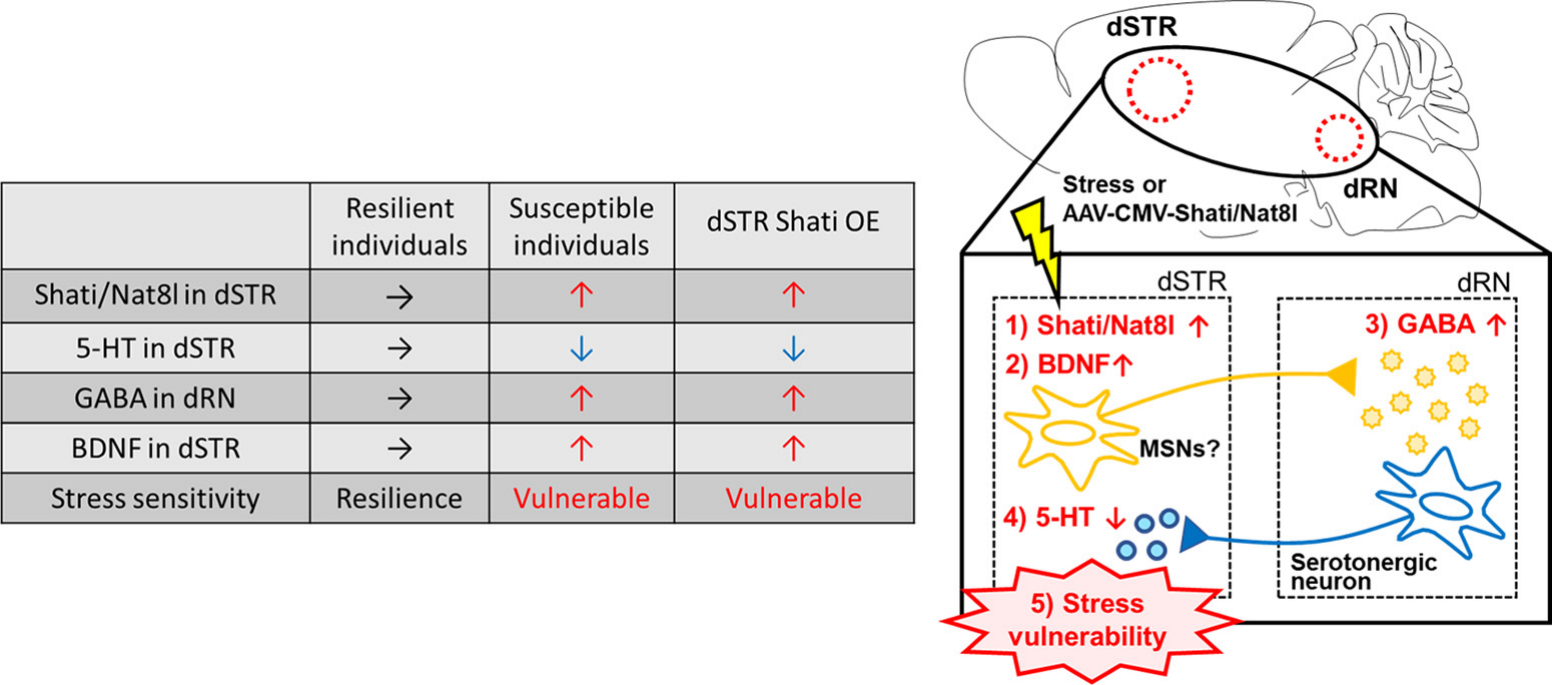
Hypothesized mechanism of establishment of stress vulnerability after RSDS. Overview of dSTR-dRN circuitry changes in the mouse brain after RSDS. Shati/Nat8l in the dSTR was increased by RSDS, and BDNF expression in the dSTR and GABA release in the dRN was increased. The serotonergic system in the dRN is deactivated by elevated GABA content, and the 5-HT content in the dSTR is decreased. Finally, stress vulnerability is established, leading to depression-like behaviors in response to stress.

In conclusion, we offer evidence supporting the role of dSTR 5-HT deficits in the pathogenesis of depression. We demonstrated that dSTR 5-HT and dRN GABA contents were regulated by dSTR Shati/Nat8l, and that these neurotransmitters contribute to stress sensitivity. Our results suggests that bidirectional dSTR-dRN neural circuitry determines stress resilience. Upregulation of dSTR 5-HT levels induces stress resilience, and overexpression of dSTR Shati/Nat8l results in enhanced BDNF expression in the dSTR and GABA release in the dRN and establishment of stress vulnerability through the reduction of 5-HT release in the dSTR (Fig. 7). Our study provides insights into the mechanism regulating stress sensitivity, a major contributor to depression onset, and indicate that targeting dSTR Shati/Nat8l allows direct regulation of the dRN serotonergic system. This may provide a novel therapeutic strategy for depression.

## Synthesis

Reviewing Editor: Christie Fowler, University of California Irvine Decisions are customarily a result of the Reviewing Editor and the peer reviewers coming together and discussing their recommendations until a consensus is reached. When revisions are invited, a fact-based synthesis statement explaining their decision and outlining what is needed to prepare a revision will be listed below. The following reviewer(s) agreed to reveal their identity: Joseph Cheer. Note: If this manuscript was transferred from JNeurosci and a decision was made to accept the manuscript without peer review, a brief statement to this effect will instead be what is listed below.

Both reviewers appreciated the changes to the manuscript and felt the data presented have been strengthened. It was noted that the inclusion of the experiment using the GABAB antagonist, CGP36216, was a strength. The results of this particular experiment are supportive of the hypothesis, and the implied bidirectional communication between dRN 5-HT neurons and dSTR GABA neurons is more plausible with the inclusion of this pharmacological experiment. However, some issues remain, which should be addressed in a revision.

1. The GABAB antagonist was very briefly mentioned in the discussion, but the results of this experiment and its implications were not discussed. A simple outline of how GABA receptor inhibition in the dRN could reduce the activity of serotonergic neurons projecting to the dSTR, and how the use of CGP36216 supported this hypothesis would be sufficient.

2. Since the viability of this proposed bidirectional mechanism hinges on an intermediary in the dSTR that is suggested to be BDNF, the unpublished figure that was shared with the reviewers (e.g., showing an increase in BDNF mRNA due to Shati/Nat8l overexpression) should be included in the manuscript. Even as a supplementary figure, it provides more credibility to the argument than citing unpublished data that isn’t shown.

3. Most of the improper grammar in the paper was addressed, and the readability of the manuscript is vastly improved from the first submission. Two minor issues that remain are as follows:

A. Page 21, line 12-14: We found that after exposure to RSDS, the basal GABA content in the dRN was higher in stress-susceptible mice, but not in resilient mice, than in naïve mice. This is a run-on sentence that feels out of order, and could be rephrased for clarity. It could be more clearly written as follows - ‘We found that after exposure to RSDS, stress-susceptible mice exhibited significantly higher basal GABA concentrations than in naive mice. This difference was not present between stress-resilient and naive mice.’

B. Page 29 lines 5-7: We demonstrated the contribution of dSTR 5-HT and dRN GABA, regulated by dSTR Shati/Nat8l, to stress sensitivity, suggesting that bidirectional dSTR-dRN neural circuitry determines stress resilience. There are similar issues with this sentence as the one above, it is a run-on sentence that could be rearranged for clarity.

## Author Response

Reviewer 1

Major comments:

1) In my view, the most exciting aspect of the study is the novel observation that Shati/Nat8l overexpression in the dorsal striatum leads to an increase in GABAergic content in the dorsal raphe. The projection from the striatum back to raphe has not been studied very much at all. This data indicates that overexpression of Shat/Nat8l somehow elevates the release or content of GABA in MSNs projecting back to the DRN. While the correlation between DRN GABA content and stress susceptibility is compelling, it does not show causation. The paper would be greatly strengthened if they performed a similar experiment as in Figure 3, except use chemogenetic inhibition of MSNs (most likely D1-MSNs) combined with DCZ infusion into the DRN. I know this is not a trivial experiment, but it would greatly enhance the paper.

Response

Thank you for the comment. Exactly, we could not get direct evidence of the function of GABA in dRN to stress sensitivity although we just conform the correlation between GABA content and social interaction ability, and we need to demonstrate that evidence of function following your advice. If we can show the contribution of dRN GABA to stress sensitivity, the findings of our study would be significantly strengthened. We considered that inhibition of dRN GABA signaling by microinfusion of CGP36216 (GABA(B) receptor antagonist) into the dRN was sufficient to demonstrate the causation between dRN GABA and stress susceptibility in dSTR Shati OE mice. In fact, we could show that inhibition of GABA signaling in the dRN induced stress resilience in dSTR-Shati OE mice. The reason why we focused on the GABA(B) is described in discussion section (line18 of page25 to line4 of page26). We also added the data showing that inhibition of dRN GABA(B) signaling in these mice suppressed dSTR-Shati-induced stress vulnerability as figure 5 (line3 of page21 to line4 of page22).

2) The second concern is one that I am conflicted about. I generally think it is a weakness to only use male mice in studies that examine stress, particularly in the context of modeling mood disorders, considering the extensive literature indicating clear and important sex differences in stress responsivity and mood disorder prevalence. That being said, this particular model, under normal conditions, only works in males and I have issues with the non-ethological way other investigators have tried to incorporate females in social defeat experiments (i.e. chemogenetic manipulations of VMH in resident mouse). The investigators should indicate that this study is done in male mice in the title and abstract as well as the methods. They should also add a discussion of sex differences and future directions in the discussion. I would encourage the authors to explore different stress paradigms in the future that can incorporate females such as chronic variable stress, social isolation. Alon Chen’s group has also shown that social crowding in adulthood is a female specific social stressor.

Response

As you mentioned, The incidence of depression is higher in women than in men (Altemus et al., 2014). However, I used only male mice in this study. Although RSDS protocol for female mice has been established using chemogenetic manipulations (Takahashi et al., 2017), male mice were used in this study because microdefeat stress requires reasonable aggressiveness of the utilized ICR mice, and I could not confirm whether the aggressivity of ICR mice to female mice in microdefeat stress is valid or not. Furthermore, we also would like to investigate the role of the dSTR-dRN neural circuitry in stress sensitivity without considering the possible influence of the estrous cycle. Therefore, male mice were used to be exposed by the validated social stress by ICR mice and prevent the effects induced by estrous cycle in depression pathogenesis. However, I understand that an experiment including female mice should be considered in future studies using appropriate protocols of social defeat stress or other female-specific social stress models, such as social crowding stress (Furman et al., 2022), because of the need to develop treatments for all patients with depression. We have added extra sentences in the discussion section and have added the references in our manuscript (line5 to 13 of page24). We also described that this study is done in male mice in the title, abstract and the method.

We cited previous study referred as reference as below

Altemus M, Sarvaiya N, Neill Epperson C (2014) Sex differences in anxiety and depression clinical perspectives. Front Neuroendocrinol 35:320-330.

Takahashi A, Chung JR, Zhang S, Zhang H, Grossman Y, Aleyasin H, Flanigan ME, Pfau ML, Menard C, Dumitriu D, Hodes GE, McEwen BS, Nestler EJ, Han MH, Russo SJ (2017) Establishment of a repeated social defeat stress model in female mice. Sci Rep 7:12838.

Furman O, Tsoory M, Chen A (2022) Differential chronic social stress models in male and female mice. Eur J Neurosci 55:2777-2793.

Minor Comments:

1) Figure 2: Make the DAPI brighter - it is difficult to see or switch it to grey scale. Also include a high-power image of the expression/fluorescence to go along with the low power image.

Response

Thank you for the comment. I made the DAPI brighter and provided high-power image in the dorsal raphe nucleus in figure 2b.

2) Figure 3: Include a high-power image to go along with the low power image in Figure 3a. For the graph labeling, I would “+” signs instead of “x” signs to indicate that these two drugs were administered together..

Response

I provided high-power image in the dorsal striatum in figure 3a. I also revised “x” signs to “+” signs.

3) There needs to be more precise information included in the viral vector descriptions. What is “mock”, is it just a eYFP control virus. Include the serotype information for all the viral vectors. Was everything AAV2/9? Typically, viral constructs only use dashes, not slashes.

Response

Im sorry, I confused you. I revised the explanation of viral vectors. The serotype of AAV-CMV-Shati/Nat8l and AAV-CMV-Mock vector for microinjection of dSTR is AAV2/9. The serotype of AAV-pet1-hM3Dq and AAV-pet1-Mock vector for microinjection of dRN is AAV2/rh10. AAV-CMV-Mock vector (AAV2/9) contained CMV promoter and cDNA encoding EGFP sequence instead of Shati/Nat8l sequence. AAV-CMV-Shati/Nat8l or Mock vectors was microinjected in dSTR, and AAV-CMV-Mock vectors were used as control for AAV-CMV-Shati/Nat8l (line12 to 15 of page8). AAV-pet1-Mock vector (AAV2/rh10) contained pet1 promoter and cDNA encoding ChR2-EYFP sequence instead of hM3Dq sequence. AAV-pet1-hM3Dq or Mock vector contained the pet1 promoter and cDNA encoding either hM3Dq or ChR2::EYFP sequence, respectively. AAV-pet1-hM3Dq or Mock vector were microinjected in dRN, and the AAV-pet1-Mock vectors were used as control for the AAV-pet1-hM3Dq (line17 of page8 to line1 of page9). I used “-”, not “/” for describing viral constructs in the text.

4) I would call the dSTR-Shati mice “dSTR-Shat OE” mice to indicate they are overexpressors

Response

Thank you for advice. I revised to “dSTR-Shati OE” from “dSTR-Shati” in the text.

5) The paper needs to be edited for clarity. There are a few places that were confusing or did not use full sentences. In the abstract, “We also assessed the contribution of GABA in modulating the serotonergic system in the dRN. GABA content in the dRN increases in the percentage of stress-susceptible mice employed produced by RSDS and dSTR-Shati OE, and is correlated with depression-like behaviors.” The sentence could also be broken up into two sentences. On page 21, “dSTR-Shati mice exhibited vulnerability to social” - should be ...to social defeat stress”. These are just two examples. The paper, overall, needs to be edited for these issues.

Response

Thank you for the comment. We exactly need to edited the paper for clarity and to improve our English. I revised these two sentences (line11 to 13 of page3, line18 of page20) you pointed, and we use reputable English language editing service (https://www.editage.com) for revising overall paper.-

Reviewer 2

Major Concerns:

1) Although the hypothesis of a bidirectional dSTR-dRN neural circuit involved in the regulation of stress vulnerability is enticing, the authors cannot infer it solely by correlating basal concentrations of GABA in the dRN with social interaction ratio (IR) and Shati/Nat8l expression. In order to imply a causal relationship between these events, authors should perform at least one more experiment to provide more robust support for their hypothesis. For example, either of the following experiments could demonstrate that modulation of GABA neurons in this pathway predictably affects depressive phenotypes: a) Intracerebral administration of a GABA antagonist in the dRN of dSTR-Mock and dSTR-Shati mice before microdefeat stress. b) dSTR injection of a GABA-targeted vector expressing hM4Di receptors, and intra-dRN administration of DCZ prior to microdefeat stress in dSTR-Shati and Mock mice.

Response

Thank you for the comment. Your critical point greatly enhances my results. Exactly, we could not get direct evidence of the function of GABA in dRN to stress sensitivity although we just conform the correlation between GABA content and social interaction ability. We performed the administration of CGP36216 (GABA(B) receptor antagonist) into the dRN to inhibit dRN GABA signaling and assessed the causation between dRN GABA and stress susceptibility in dSTR Shati OE mice following your advice. In fact, we could show that inhibition of GABA signaling in the dRN induced stress resilience in dSTR-Shati OE mice. The reason why we focused on the GABA(B) is described in discussion section (line18 of page25 to line4 of page26). We also added the data showing that inhibition of dRN GABA(B) signaling in these mice suppressed dSTR-Shati-induced stress vulnerability as figure 5 (line3 of page21 to line4 of page22).

2) The manuscript should also include additional discussion to explain the proposed mechanistic relationship between high levels of Shati/Nat8l in the dSTR and activation of GABAergic neurons projecting to the dRN.

Response

Thank you for the comment. I added the discussion about the contribution of dSTR-Shati/Nat8l to activation of GABAergic neurons projecting to the dRN (line17 of page26 to line12 of page 27). One possible regulatory mechanism is BDNF. dSTR Shati/Nat8l knockdown mice exhibit reduced BDNF mRNA and protein levels in the dSTR and resilience to high-intensity RSDS exposure (Miyanishi and Nitta, 2021). Furthermore, inhibition of BDNF signaling by ANA-12 (a tyrosine protein kinase inhibitor) induces stress resilience (Miyanishi et al., 2021). We confirmed that dSTR Shati/Nat8l OE mice have higher BDNF mRNA levels in the dSTR compared to those in mock mice (unpublished data) (the data are below), and this result is consisted with our previous study. BDNF promotes neurogenesis, neuronal excitability, and plasticity by exerting neurotrophic functions (Ferrini and De Koninck, 2013; Colucci-D’Amato et al., 2020; Yang et al., 2020). In the present study, we observed an increase in dRN-GABA content in stress-susceptible and dSTR-Shati OE mice. The activation of GABAergic neurons projecting from the dSTR to the dRN may be induced by the upregulation of striatal BDNF levels.

I cited previous study referred in the text as below

Miyanishi H, Muramatsu SI, Nitta A (2021) Striatal Shati/Nat8l-BDNF pathways determine the sensitivity to social defeat stress in mice through epigenetic regulation. Neuropsychopharmacology 46:1594-1605.

Miyanishi H, Nitta A (2021) A role of BDNF in the depression pathogenesis and a potential target as antidepressant: the modulator of stress sensitivity “Shati/Nat8l-BDNF system” in the dorsal striatum. Pharmaceuticals (Basel) 14:889.

Ferrini F, De Koninck Y (2013) Microglia control neuronal network excitability via BDNF signalling. Neural Plast 2013:429815.

Colucci-D’Amato L, Speranza L, Volpicelli F (2020) Neurotrophic factor BDNF, physiological functions and therapeutic potential in depression, neurodegeneration and brain cancer. Int J Mol Sci 21:7777.

Yang T, Nie Z, Shu H, Kuang Y, Chen X, Cheng J, Yu S, Liu H. (2020) The role of BDNF on neural plasticity in depression. Front Cell Neurosci 14:82.

Data are expressed as mean {plus minus} standard error of the mean (SEM)

dSTR-Mock, n = 6; dSTR-Shati OE, n = 5; *p < 0.05 vs dSTR-Mock mice (student t-test)

3) The structure, organization, and faulty grammar found throughout this paper need to be addressed. The methods section needs to be re-structured for clarity. The disorganized nature of the writing here makes it unclear which surgeries were performed in each experiment, what exact viral constructs were used, and how microinfusions were conducted. One such example is the use of a vector containing ChR2-eYFP to label serotonin neurons of the dRN in Mock animals. In addition to it being unclear which experiments ChR2-eYFP was used in, no optical stimulation was performed in this experiment and no controls for light within the activation wavelength of ChR2 were discussed.

Response

I’m sorry that my english skill is not good. I confirmed the structure and organization in this paper, then, and we use reputable English language editing service (https://www.editage.com) for revising overall paper. We described about surgeries in each experiments, viral constructs used each experiments and microinfusions methods in methods section.

Surgery of guide cannula implantation were performed for microinfusion of DCZ or CGP36216 (line15 to 18 of page13) and In vivo microdialysis (line10 to 11 of page14). AAV-CMV-Mock vector (AAV2/9) contained CMV promoter and cDNA encoding EGFP sequence instead of Shati/Nat8l sequence. AAV-CMV-Shati/Nat8l or Mock vectors was microinjected in dSTR, and AAV-CMV-Mock vectors were used as control for AAV-CMV-Shati/Nat8l (line12 to 15 of page8). AAV-pet1-Mock vector (AAV2/rh10) contained pet1 promoter and cDNA encoding ChR2-EYFP sequence instead of hM3Dq sequence. AAV-pet1-hM3Dq or Mock vector contained the pet1 promoter and cDNA encoding either hM3Dq or ChR2::EYFP sequence, respectively. AAV-pet1-hM3Dq or Mock vector were microinjected in dRN, and the AAV-pet1-Mock vectors were used as control for the AAV-pet1-hM3Dq (line17 of page8 to line1 of page9). In microinfusion, drugs　(DCZ and CGP36216 (GABA(B) receptor antagonist)) were infused by inserting the broken dialysis probe (A-I-4-01, Eicom) through the guide cannula implanted in mice brain using an injector EPS-64 micro syringe pump (Eicom) (line18 of page13 to line3 of page14).

As you mentioned, although ChR2 was used for the optogenetic experiments, I did not perform that experiment in this paper. I just used AAV-pet1-Mock vector encoding ChR2-EYFP sequence as control for AAV-pet1-hM3Dq vector. I considered that it is no problem to use AAV-pet1-Mock vector as control because previous study showed that expression of ChR2 did not affect the behaviors without optogenetic stimulation (Challis et al., 2014; Morel et al., 2022).

I cited previous study referred in here as below

Challis C, Beck SG, Berton O (2014) Optogenetic modulation of descending prefrontocortical inputs to the dorsal raphe bidirectionally bias socioaffective choices after social defeat. Front Behav Neurosci 8:43.

Morel C et al. (2022) Midbrain projection to the basolateral amygdala encodes anxiety-like but not depression-like behaviors. Nat Commun. 13:1532.

4) The DREADD manipulation of dRN-dSTR serotonergic neurons during micro-defeat stress was intriguing, but it would be important to understand the impact of this technique in RSDS as well. The authors also could have shown how DREADD-mediated activation of the dRN-dSTR pathway would impact behavioral measurements in the RSDS to establish a comparison with the micro-defeat model.

Response

Thank you for the comment. In this study, I just used microdefeat stress, but not RSDS, for behavioral assessment of dSTR-Shati OE mice. RSDS induced depression-like behaviors in both of control mice (Mock) and dSTR shati OE mice. In order to investigate the difference of stress sensitivity in these mice, we needed to use the validated stress model that do not affect to behaviors of control mice. If we used RSDS following your advice, we can apply to wildtype mice with DREADD activation of dRN-dSTR serotonergic neurons, and this experiment of RSDS might provide a good discussion. However, in this study, we wanted to demonstrate that regulation of dRN-dSTR serotonergic neurons is mediated by dSTR-Shati/Nat8l. Therefore, we considered that our experiment of microdefeat stress is enough for showing the involvement of Shati/nat8l to stress sensitivity.

5) Considering that direct serotoninergic activation elicits a rapid release of 5-HT, how would the authors explain that 5-HT content increase in the dSTR occurred only after 36 minutes of the pharmacological activation of dRN serotoninergic neurons?

Response

Thank you for the comment. Honestly, I also do not fully understand that 5-HT content increase in the dSTR occurred only 36 minutes after microinfusion of DCZ. Technically or physiologically factor might explain the phenomenon. However, most important thing is to match the timing of upregulation of dSTR 5-HT and microdefeat stress. I planned time schedule of microdefeat stress for matching to timing of 5-HT increase, and detailly performed. Therefore, I considered that the results in this study could demonstrate the relationship between dSTR 5-HT and stress sensitivity, and my contention is not changed.

6) It is not clear at what point in time the basal content of GABA and 5HT are measured and what were the experimental conditions for it.

Response

Thank you for the comment. I’m sorry that I did not describe detailed information regarding basal content of 5-HT and GABA.

It takes about more than 2 h until 5-HT and GABA stabilize. I observed the change of 5-HT levels in live, and defined the baseline 5-HT levels as the average of four consecutive fractions (the difference between each value was less than 10%). In GABA measurement, I cannot observe the alteration of GABA level in live because of machine system. I considered that GABA levels is definitely stable 4 h after probe insertion, and actually confirmed that. Thus, the average of four consecutive dialysates collected 4 h after probe insertion was defined as the baseline GABA level. I described this information regarding baseline of 5-HT and GABA in the method section (line14 to 16 of page 14, line6 to 7 of page15).

Minor Concerns:

1) In the introduction, the description of major depressive disorder is confusing and lacks recent epidemiological data which can be found accessing the Institute of Health Metrics and Evaluation. Global Health Data Exchange (GHDx). https://vizhub.healthdata.org/gbd-results/.

Response

Thank you for advice. I referred the recent data of GBD study regarding the increase in number of depression patients (GBD 2019 Mental Disorders Collaborators, 2022) which can be found accessing the Institute of Health Metrics and Evaluation in introduction (line3 to 4 of page5).

I cited previous study referred in the text as below

GBD 2019 Mental Disorders Collaborators (2022) Global, regional, and national burden of 12 mental disorders in 204 countries and territories, 1990-2019: a systematic analysis for the Global Burden of Disease Study 2019. Lancet Psychiatry 9:137-150.

2) The discussion section starts by rephrasing the manuscript findings and justifying why the authors chose to explore dSTR-dRN pathway. The same information is explained again after only two paragraphs, causing redundancy that should be avoided.

Response

We have deleted same information of the second paragraph in the discussion section.

3) The studies cited as providing evidence of stress as a major environmental risk factor for depression are also not the ones with this information. Maybe a better reference would be (doi:10.1176/ajp.156.6.837)

Response

Thank you for teaching the interesting reference. I referred the article in introduction section (line13 to 14 of page5).

I cited previous study referred in the text as below

Kendler KS, Karkowski LM, Prescott CA (1999) Causal relationship between stressful life events and the onset of major depression. Am J Psychiatry. 156:837-841.

4) Benmansour et al., 1999 does not evaluate stress response so the sentence referring to it must be rephrased.

Response

Thank you for the comment. I wanted to describe that control of 5-HT levels by SSRI treatment involved in stress response using reference. As you mentioned, however, Benmansour et al did not explain about that. Therefore, the citation of Benmansour et al was deleted. Instead of the deleted sentence, I explained the relationship between stress sensitivity and 5-HT increase by SSRI treatment using other reference (Uno et al., 2019) (line 9 of page6).

I cited previous study referred in the text as below

Uno K, Miyanishi H, Sodeyama K, Fujiwara T, Miyazaki T, Muramatsu SI, Nitta A (2019) Vulnerability to depressive behavior induced by overexpression of striatal Shati/Nat8l via the serotonergic neuronal pathway in mice. Behav Brain Res 376:112227.

5) When describing a well-known neurobiology event (synthesis of 5HT by tryptophan hydroxylase) the authors should not cite a recent article (Cheng et al., 2020) that does not provide new proof of this already established mechanism.

Response

Thank you for the comment. I deleted the recent article (Cheng et al., 2020) from the manuscript.

6) In-text reference to Miyamoto et al., 2003 appears to have been published in 2017.

Response

I revised that (line14 of page6).

7) Miyashini et al., published two articles in 2021 and there is no distinction between them when cited in-text (2021a / 2021b could discern between the two, or include the second author before et al.).

Response

Thank you for the advice. I described first and second author (Miyanishi and Nitta, 2021) in the text when there are just two authors in the article. On the other hand, when the article has multiple authors, I described as “Miyanishi et al., 2021” in the text.

8) The use of both interaction zone (IZ) time and social interaction ratio (IR) in independent graphs in the figures is slightly redundant considering that IR is the IZ post-test adjusted by IZ pre-test.

Response

Thank you for the comment. As you said, both graph of IZ time and IR time show the same interpretation. However, while the result of IR depends on pre-test in social interaction test, IZ time depends on post-test in social interaction test. I showed IR and IZ graph following the previous article (Golden et al., 2011) in this study, and I considered that two result including IZ time and IR is needed to show validity and completion of RSDS and social interaction test.

I cited previous study referred in here as below

Golden SA, Covington HE 3rd, Berton O, Russo SJ (2011) A standardized protocol for repeated social defeat stress in mice. Nat Protoc 6:1183-1191.

9) The choice of 36B4 as a housekeeping gene should be mentioned in the methods section and if possible, the results to it should be shown in the figure or supplemental data.

Response

Thank you for the comment. I added the sentence regarding the use of 36B4 as a housekeeping in the method section (line8 to 9 of page10). 36B4 were used as a housekeeping gene in many previous articles, and I actually confirmed that overexpression of dSTR-Shati/Nat8l did not affect 36B4 expression.

10) Sequences for RT-PCR primers used ideally should be supplied and not cited. If there is no space available in the manuscript, provide it as supplementary table.

Response

Thank you for the advice. I added the information of sequence of primer in the method section (line11 to 16 of page10).

11) Statistical analysis lacked information regarding normality testing prior to ANOVA use and what was done if data distribution was not normal.

Response

In all experiments in this study, obtained data are predicted to show normal distribution. Thus, parametric statistical tests were applied. In this journal “eNeuro”, paper with the similar statistical procedures were frequently published (Sung et al., 2023; Okamura et al., 2022).

I cited previous study referred in here as below.

Sung JH, Ou Y, Barger SW. (2023) Amyloid β-Peptide Effects on Glucose Regulation Are Dependent on Apolipoprotein E Genotype. eNeuro 10:ENEURO.0376-22.2023.

Okamura H, Yasugaki S, Suzuki-Abe H, Arai Y, Sakurai K, Yanagisawa M, Takizawa H, Hayashi Y (2022) Long-Term Effects of Repeated Social Defeat Stress on Brain Activity during Social Interaction in BALB/c Mice. eNeuro 9:ENEURO.0068-22.2022.

12) Authors should not cite their own articles in every method section when providing details of the protocol, especially regarding methodologies that were well-established by other scientists (i.e. immunostaining).

Response

Thank you for the advice. I referred other article regarding immunostaining, sucrose preference test, locomotor activity test, tail suspension test and forced swimming test in the methods section instead of our article.

I cited previous study referred in the text as below.

Golden SA, Covington HE 3rd, Berton O, Russo SJ (2011) A standardized protocol for repeated social defeat stress in mice. Nat Protoc 6:1183-1191.

Ibi D, Takuma K, Koike H, Mizoguchi H, Tsuritani K, Kuwahara Y, Kamei H, Nagai T, Yoneda Y, Nabeshima T, Yamada K (2008) Social isolation rearing-induced impairment of the hippocampal neurogenesis is associated with deficits in spatial memory and emotion-related behaviors in juvenile mice. J Neurochem 105:921-932.Ferrini F, De Koninck Y (2013) Microglia control neuronal network excitability via BDNF signalling. Neural Plast 2013:429815.

Ma M, Ren Q, Yang C, Zhang JC, Yao W, Dong C, Ohgi Y, Futamura T, Hashimoto K (2017) Antidepressant effects of combination of brexpiprazole and fluoxetine on depression-like behavior and dendritic changes in mice after inflammation. Psychopharmacology (Berl) 234:525-533.

Machado DG, Bettio LE, Cunha MP, Santos AR, Pizzolatti MG, Brighente IM, Rodrigues AL (2008) Antidepressant-like effect of rutin isolated from the ethanolic extract from Schinus molle L. in mice: evidence for the involvement of the serotonergic and noradrenergic systems. Eur J Pharmacol 587:163-168.

Zhang K, Fujita Y, Hashimoto K (2018) Lack of metabolism in (R)-ketamine’s antidepressant actions in a chronic social defeat stress model. Sci Rep 8:4007.

13) In figure 3j the Y-axis mentions the following unit: locomotor activity (counts/60min) x 103. It is not clear to what this unit refers to. Additional information could be added in method session or figure legend.

Response

I’m sorry that I confused you. I added the information about the unit (count) in the method section (line19 of page12 to line1 of page13). According to guide manual, the infrared sensor is arranged in SCANET MV-40. Locomotor activity, measured as “counts”, increased when the mice passed a set of infrared beams in SCANET MV-40.

14) In the “Striatal Shanti/Nat8l expression and vulnerability to stress” results section, authors refer to figures 2i and 2j when they are referring to 3i and 3j respectively.

Response

I revised that (line13 of page 19, line17 of page19).

15) Manuscript title does not convey the most interesting findings of this work and rephrases results already published by this group. I would suggest a new title that mentions the role of GABA in the dSTR-dRN circuit regulating stress vulnerability in mice with high levels of Shati/Nat8l.

Response

Thank you for comment. I revised title to “The role of GABA in the in the dorsal striatum-raphe nucleus circuit regulating stress vulnerability in male mice with high levels of Shati/Nat8l.” following your advice.

16) In the first page of the results section authors refer to microdialysis as microanalysis. This should be corrected.

Response

I revised that (line14 of page16).

## References

[B1] Alexander RC (2017) The potential efficacy of GABAB antagonists in depression. Curr Opin Pharmacol 35:101–104. 10.1016/j.coph.2017.07.009 28807483

[B2] Altemus M, Sarvaiya N, Neill Epperson C (2014) Sex differences in anxiety and depression clinical perspectives. Front Neuroendocrinol 35:320–330. 10.1016/j.yfrne.2014.05.004 24887405 PMC4890708

[B3] Amemori KI, Amemori S, Gibson DJ, Graybiel AM (2018) Striatal microstimulation induces persistent and repetitive negative decision-making predicted by striatal beta-band oscillation. Neuron 99:829–841.e6. 10.1016/j.neuron.2018.07.022 30100255 PMC6269092

[B4] Ariyannur PS, Moffett JR, Manickam P, Pattabiraman N, Arun P, Nitta A, Nabeshima T, Madhavarao CN, Namboodiri AM (2010) Methamphetamine-induced neuronal protein NAT8L is the NAA biosynthetic enzyme: implications for specialized acetyl coenzyme a metabolism in the CNS. Brain Res 1335:1–13. 10.1016/j.brainres.2010.04.008 20385109

[B5] Asaoka N, Nishitani N, Kinoshita H, Kawai H, Shibui N, Nagayasu K, Shirakawa H, Nakagawa T, Kaneko S (2017) Chronic antidepressant potentiates spontaneous activity of dorsal raphe serotonergic neurons by decreasing GABAB receptor-mediated inhibition of L-type calcium channels. Sci Rep 7:13609. 10.1038/s41598-017-13599-3 29051549 PMC5648823

[B6] Azmitia EC, Segal M (1978) An autoradiographic analysis of the differential ascending projections of the dorsal and median raphe nuclei in the rat. J Comp Neurol 179:641–667. 10.1002/cne.901790311 565370

[B7] Babenko VN, Galyamina AG, Rogozin IB, Smagin DA, Kudryavtseva NN (2020) Dopamine response gene pathways in dorsal striatum MSNs from a gene expression viewpoint: cAMP-mediated gene networks. BMC Neurosci 21:12. 10.1186/s12868-020-00560-w 32216748 PMC7099774

[B8] Belleau EL, Treadway MT, Pizzagalli DA (2019) The impact of stress and major depressive disorder on hippocampal and medial prefrontal cortex morphology. Biol Psychiatry 85:443–453. 10.1016/j.biopsych.2018.09.031 30470559 PMC6380948

[B9] Bourke CH, Glasper ER, Neigh GN (2014) SSRI or CRF antagonism partially ameliorate depressive-like behavior after adolescent social defeat. Behav Brain Res 270:295–299. 10.1016/j.bbr.2014.05.035 24867331

[B10] Brady ML, Pilli J, Lorenz-Guertin JM, Das S, Moon CE, Graff N, Jacob TC (2018) Depolarizing, inhibitory GABA type A receptor activity regulates GABAergic synapse plasticity via ERK and BDNF signaling. Neuropharmacology 128:324–339. 10.1016/j.neuropharm.2017.10.022 29074304 PMC5739058

[B11] Carnevali L, Montano N, Tobaldini E, Thayer JF, Sgoifo A (2020) The contagion of social defeat stress: insights from rodent studies. Neurosci Biobehav Rev 111:12–18. 10.1016/j.neubiorev.2020.01.011 31931035

[B12] Chaki S, Fukumoto K (2019) Role of serotonergic system in the antidepressant actions of mGlu2/3 receptor antagonists: similarity to ketamine. Int J Mol Sci 20:1270. 10.3390/ijms20061270 30871246 PMC6470808

[B13] Colucci-D’Amato L, Speranza L, Volpicelli F (2020) Neurotrophic factor BDNF, physiological functions and therapeutic potential in depression, neurodegeneration and brain cancer. Int J Mol Sci 21:7777. 10.3390/ijms21207777 33096634 PMC7589016

[B14] Cox J, Witten IB (2019) Striatal circuits for reward learning and decision-making. Nat Rev Neurosci 20:482–494. 10.1038/s41583-019-0189-2 31171839 PMC7231228

[B15] Dell'Osso L, Carmassi C, Mucci F, Marazziti D (2016) Depression, serotonin and tryptophan. Curr Pharm Des 22:949–954. 10.2174/1381612822666151214104826 26654774

[B16] Dudek KA, Dion-Albert L, Kaufmann FN, Tuck E, Lebel M, Menard C (2021) Neurobiology of resilience in depression: immune and vascular insights from human and animal studies. Eur J Neurosci 53:183–221. 10.1111/ejn.14547 31421056 PMC7891571

[B17] Ferrini F, De Koninck Y (2013) Microglia control neuronal network excitability via BDNF signalling. Neural Plast 2013:429815. 10.1155/2013/429815 24089642 PMC3780625

[B18] Fleshner M, Maier SF, Lyons DM, Raskind MA (2011) The neurobiology of the stress-resistant brain. Stress 14:498–502. 10.3109/10253890.2011.596865 21790482 PMC3287388

[B19] Fu K, Lin H, Miyamoto Y, Wu C, Yang J, Uno K, Nitta A (2016) Pseudoginsenoside-F11 inhibits methamphetamine-induced behaviors by regulating dopaminergic and GABAergic neurons in the nucleus accumbens. Psychopharmacology (Berl) 233:831–840. 10.1007/s00213-015-4159-8 26621348

[B20] Fukumoto K, Iijima M, Chaki S (2016) The antidepressant effects of an mGlu2/3 receptor antagonist and ketamine require AMPA receptor stimulation in the mPFC and subsequent activation of the 5-HT neurons in the DRN. Neuropsychopharmacology 41:1046–1056. 10.1038/npp.2015.233 26245499 PMC4748429

[B21] Fukumoto K, Iijima M, Funakoshi T, Chaki S (2018) Role of 5-HT1A receptor stimulation in the medial prefrontal cortex in the sustained antidepressant effects of ketamine. Int J Neuropsychopharmacol 21:371–381. 10.1093/ijnp/pyx116 29309585 PMC5888010

[B22] Furman O, Tsoory M, Chen A (2022) Differential chronic social stress models in male and female mice. Eur J Neurosci 55:2777–2793. 10.1111/ejn.15481 34587653

[B23] GBD 2019 Mental Disorders Collaborators (2022) Global, regional, and national burden of 12 mental disorders in 204 countries and territories, 1990-2019: a systematic analysis for the Global Burden of Disease Study 2019. Lancet Psychiatry 9:137–150. 10.1016/S2215-0366(21)00395-3 35026139 PMC8776563

[B24] Golden SA, Covington HE 3rd, Berton O, Russo SJ (2011) A standardized protocol for repeated social defeat stress in mice. Nat Protoc 6:1183–1191. 10.1038/nprot.2011.361 21799487 PMC3220278

[B25] Graeff FG, Guimarães FS, De Andrade TG, Deakin JF (1996) Role of 5-HT in stress, anxiety, and depression. Pharmacol Biochem Behav 54:129–141. 10.1016/0091-3057(95)02135-3 8728550

[B26] Graveland GA, DiFiglia M (1985) The frequency and distribution of medium-sized neurons with indented nuclei in the primate and rodent neostriatum. Brain Res 327:307–311. 10.1016/0006-8993(85)91524-0 3986508

[B27] Haddar M, Uno K, Azuma K, Muramatsu SI, Nitta A (2020) Inhibitory effects of Shati/Nat8l overexpression in the medial prefrontal cortex on methamphetamine‐induced conditioned place preference in mice. Addict Biol 25:e12749. 10.1111/adb.12749 30950164 PMC7187255

[B28] Hale MW, Shekhar A, Lowry CA (2012) Stress-related serotonergic systems: implications for symptomatology of anxiety and affective disorders. Cell Mol Neurobiol 32:695–708. 10.1007/s10571-012-9827-1 22484834 PMC3378822

[B29] Hasegawa E, Yanagisawa M, Sakurai T, Mieda M (2014) Orexin neurons suppress narcolepsy via 2 distinct efferent pathways. J Clin Invest 124:604–616. 10.1172/JCI71017 24382351 PMC3904620

[B30] Hauenstein EJ (2003) Depression in adolescence. J Obstet Gynecol Neonatal Nurs 32:239–248. 10.1177/0884217503252133 12685676

[B31] Heshmati M, Christoffel DJ, LeClair K, Cathomas F, Golden SA, Aleyasin H, Turecki G, Friedman AK, Han MH, Menard C, Russo SJ (2020) Depression and social defeat stress are associated with inhibitory synaptic changes in the nucleus accumbens. J Neurosci 40:6228–6233. 10.1523/JNEUROSCI.2568-19.2020 32561672 PMC7406280

[B32] Hofmann SG, Curtiss J, Carpenter JK, Kind S (2017) Effect of treatments for depression on quality of life: a meta-analysis. Cogn Behav Ther 46:265–286. 10.1080/16506073.2017.1304445 28440699 PMC5663193

[B33] Hornung JP (2003) The human raphe nuclei and the serotonergic system. J Chem Neuroanat 26:331–343. 10.1016/j.jchemneu.2003.10.002 14729135

[B34] Ibi D, Takuma K, Koike H, Mizoguchi H, Tsuritani K, Kuwahara Y, Kamei H, Nagai T, Yoneda Y, Nabeshima T, Yamada K (2008) Social isolation rearing-induced impairment of the hippocampal neurogenesis is associated with deficits in spatial memory and emotion-related behaviors in juvenile mice. J Neurochem 105:921–932. 10.1111/j.1471-4159.2007.05207.x 18182044

[B35] Iida A, Takino N, Miyauchi H, Shimazaki K, Muramatsu S (2013) Systemic delivery of tyrosine-mutant AAV vectors results in robust transduction of neurons in adult mice. Biomed Res Int 2013:974819. 10.1155/2013/974819 23762870 PMC3671507

[B36] Ishimura K, Takeuchi Y, Fujiwara K, Tominaga M, Yoshioka H, Sawada T (1988) Quantitative analysis of the distribution of serotonin-immunoreactive cell bodies in the mouse brain. Neurosci Lett 91:265–270. 10.1016/0304-3940(88)90691-x 3185964

[B37] Kang M, Pyun KH, Jang CG, Kim H, Bae H, Shim I (2005) Nelumbinis Semen reverses a decrease in hippocampal 5-HT release induced by chronic mild stress in rats. J Pharm Pharmacol 57:651–656. 10.1211/0022357056055 15901354

[B38] Kemp JM, Powell TP (1971) The structure of the caudate nucleus of the cat: light and electron microscopy. Philos Trans R Soc Lond B Biol Sci 262:383–401. 10.1098/rstb.1971.0102 4107495

[B39] Kendler KS, Karkowski LM, Prescott CA (1999) Causal relationship between stressful life events and the onset of major depression. Am J Psychiatry 156:837–841. 10.1176/ajp.156.6.837 10360120

[B40] Kessler RC, Berglund P, Demler O, Jin R, Koretz D, Merikangas KR, Rush AJ, Walters EE, Wang PS; National Comorbidity Survey Replication (2003) The epidemiology of major depressive disorder: results from the National Comorbidity Survey Replication (NCS-R). JAMA 289:3095–3105. 10.1001/jama.289.23.3095 12813115

[B41] Klawonn AM, Fritz M, Castany S, Pignatelli M, Canal C, Similä F, Tejeda HA, Levinsson J, Jaarola M, Jakobsson J, Hidalgo J, Heilig M, Bonci A, Engblom D (2021) Microglial activation elicits a negative affective state through prostaglandin-mediated modulation of striatal neurons. Immunity 54:225–234.e6. 10.1016/j.immuni.2020.12.016 33476547

[B42] Kreitzer AC (2009) Physiology and pharmacology of striatal neurons. Annu Rev Neurosci 32:127–147. 10.1146/annurev.neuro.051508.135422 19400717

[B43] Krzyzosiak A, Szyszka-Niagolov M, Wietrzych M, Gobaille S, Muramatsu S, Krezel W (2010) Retinoid x receptor gamma control of affective behaviors involves dopaminergic signaling in mice. Neuron 66:908–920. 10.1016/j.neuron.2010.05.004 20620876

[B44] Lago T, Davis A, Grillon C, Ernst M (2017) Striatum on the anxiety map: small detours into adolescence. Brain Res 1654:177–184. 10.1016/j.brainres.2016.06.006 27276526 PMC5140771

[B45] Le JJ, Yi T, Qi L, Li J, Shao L, Dong JC (2016) Electroacupuncture regulate hypothalamic-pituitary-adrenal axis and enhance hippocampal serotonin system in a rat model of depression. Neurosci Lett 615:66–71. 10.1016/j.neulet.2016.01.004 26773866

[B46] Li ZL, Wang Y, Zou HW, Jing XY, Liu YJ, Li LF (2021) GABA(B) receptors within the lateral habenula modulate stress resilience and vulnerability in mice. Physiol Behav 230:113311. 10.1016/j.physbeh.2021.113311 33412189

[B47] Liu Y, Steinhausen K, Bharwani A, Mian MF, McVey Neufeld KA, Forsythe P (2020) Increased persistence of avoidance behaviour and social deficits with L.rhamnosus JB-1 or selective serotonin reuptake inhibitor treatment following social defeat. Sci Rep 10:18501. 10.1038/s41598-020-69968-y 33097815 PMC7584642

[B48] Luo DD, An SC, Zhang X (2008) Involvement of hippocampal serotonin and neuropeptide Y in depression induced by chronic unpredicted mild stress. Brain Res Bull 77:8–12. 10.1016/j.brainresbull.2008.05.010 18579108

[B49] Ma M, Ren Q, Yang C, Zhang JC, Yao W, Dong C, Ohgi Y, Futamura T, Hashimoto K (2017) Antidepressant effects of combination of brexpiprazole and fluoxetine on depression-like behavior and dendritic changes in mice after inflammation. Psychopharmacology (Berl) 234:525–533. 10.1007/s00213-016-4483-7 27844095 PMC5263204

[B50] Machado DG, Bettio LE, Cunha MP, Santos AR, Pizzolatti MG, Brighente IM, Rodrigues AL (2008) Antidepressant-like effect of rutin isolated from the ethanolic extract from *Schinus molle* L. in mice: evidence for the involvement of the serotonergic and noradrenergic systems. Eur J Pharmacol 587:163–168. 10.1016/j.ejphar.2008.03.021 18457827

[B51] Mata DA, Ramos MA, Bansal N, Khan R, Guille C, Di Angelantonio E, Sen S (2015) Prevalence of depression and depressive symptoms among resident physicians: a systematic review and meta-analysis. JAMA 314:2373–2383. 10.1001/jama.2015.15845 26647259 PMC4866499

[B52] Mao M, Nair A, Augustine GJ (2019) A Novel type of neuron within the dorsal striatum. Front Neural Circuits 13:32. 10.3389/fncir.2019.00032 31164808 PMC6536632

[B53] Miyamoto Y, Iegaki N, Fu K, Ishikawa Y, Sumi K, Azuma S, Uno K, Muramatsu SI, Nitta A (2017) Striatal N-acetylaspartate synthetase Shati/Nat8l regulates depression-like behaviors via mGluR3-mediated serotonergic suppression in mice. Int J Neuropsychopharmacol 20:1027–1035. 10.1093/ijnp/pyx078 29020418 PMC5716104

[B54] Miyanishi H, Nitta A (2021) A role of BDNF in the depression pathogenesis and a potential target as antidepressant: the modulator of stress sensitivity “Shati/Nat8l-BDNF system” in the dorsal striatum. Pharmaceuticals (Basel) 14:889. 10.3390/ph14090889 34577589 PMC8469819

[B55] Miyanishi H, Uno K, Iwata M, Kikuchi Y, Yamamori H, Yasuda Y, Ohi K, Hashimoto R, Hattori K, Yoshida S, Goto YI, Sumiyoshi T, Nitta A (2020) Investigating DNA methylation of SHATI/NAT8L promoter sites in blood of unmedicated patients with major depressive disorder. Biol Pharm Bull 43:1067–1072. 10.1248/bpb.b19-01099 32612069

[B56] Miyanishi H, Muramatsu SI, Nitta A (2021) Striatal Shati/Nat8l-BDNF pathways determine the sensitivity to social defeat stress in mice through epigenetic regulation. Neuropsychopharmacology 46:1594–1605. 10.1038/s41386-021-01033-2 34099867 PMC8280178

[B58] Mombereau C, Kaupmann K, Froestl W, Sansig G, van der Putten H, Cryan JF (2004) Genetic and pharmacological evidence of a role for GABA(B) receptors in the modulation of anxiety- and antidepressant-like behavior. Neuropsychopharmacology 29:1050–1062. 10.1038/sj.npp.1300413 15039762

[B59] Nagai Y, et al. (2020) Deschloroclozapine, a potent and selective chemogenetic actuator enables rapid neuronal and behavioral modulations in mice and monkeys. Nat Neurosci 23:1157–1167. 10.1038/s41593-020-0661-3 32632286

[B60] Nestler EJ, Carlezon WA Jr (2006) The mesolimbic dopamine reward circuit in depression. Biol Psychiatry 59:1151–1159. 10.1016/j.biopsych.2005.09.018 16566899

[B61] Niwa M, Nitta A, Mizoguchi H, Ito Y, Noda Y, Nagai T, Nabeshima T (2007) A novel molecule “shati” is involved in methamphetamine-induced hyperlocomotion, sensitization, and conditioned place preference. J Neurosci 27:7604–7615. 10.1523/JNEUROSCI.1575-07.2007 17626222 PMC6672622

[B62] Pandarakalam JP (2018) Challenges of treatment-resistant depression. Psychiatr Danub 30:273–284. 10.24869/psyd.2018.273 30267518

[B63] Paul ED, Hale MW, Lukkes JL, Valentine MJ, Sarchet DM, Lowry CA (2011) Repeated social defeat increases reactive emotional coping behavior and alters functional responses in serotonergic neurons in the rat dorsal raphe nucleus. Physiol Behav 104:272–282. 10.1016/j.physbeh.2011.01.006 21238469 PMC3089807

[B64] Paxinos G, Franklin KBJ (2008) The mouse brain in stereotaxic coordinates, Ed 3. Amsterdam: Elsevier.

[B65] Petroff OA (2002) GABA and glutamate in the human brain. Neuroscientist 8:562–573. 10.1177/1073858402238515 12467378

[B66] Pollak DI, Fürth D, Xuan Y, Johansson Y, Pozzi L, Silberberg G, Carlén M, Meletis K (2014) A whole-brain atlas of inputs to serotonergic neurons of the dorsal and median raphe nuclei. Neuron 83:663–678. 10.1016/j.neuron.2014.07.002 25102561

[B67] Rosenblat JD, Carvalho AF, Li M, Lee Y, Subramanieapillai M, McIntyre RS (2019) Oral ketamine for depression: a systematic review. J Clin Psychiatry 80:18r12475. 10.4088/JCP.18r12475 30995364

[B68] Sachs BD, Ni JR, Caron MG (2015) Brain 5-HT deficiency increases stress vulnerability and impairs antidepressant responses following psychosocial stress. Proc Natl Acad Sci U S A 112:2557–2562. 10.1073/pnas.1416866112 25675490 PMC4345581

[B69] Takahashi A, Chung JR, Zhang S, Zhang H, Grossman Y, Aleyasin H, Flanigan ME, Pfau ML, Menard C, Dumitriu D, Hodes GE, McEwen BS, Nestler EJ, Han MH, Russo SJ (2017) Establishment of a repeated social defeat stress model in female mice. Sci Rep 7:12838. 10.1038/s41598-017-12811-8 28993631 PMC5634448

[B70] Tao R, Auerbach SB (2000) Regulation of serotonin release by GABA and excitatory amino acids. J Psychopharmacol 14:100–113. 10.1177/026988110001400201 10890306

[B71] Uno K, Miyanishi H, Sodeyama K, Fujiwara T, Miyazaki T, Muramatsu SI, Nitta A (2019) Vulnerability to depressive behavior induced by overexpression of striatal Shati/Nat8l via the serotonergic neuronal pathway in mice. Behav Brain Res 376:112227. 10.1016/j.bbr.2019.112227 31520691

[B72] Yang T, Nie Z, Shu H, Kuang Y, Chen X, Cheng J, Yu S, Liu H (2020) The role of BDNF on neural plasticity in depression. Front Cell Neurosci 14:82. 10.3389/fncel.2020.00082 32351365 PMC7174655

[B73] Yuan TF, Paes F, Arias-Carrión O, Ferreira Rocha NB, de Sá Filho AS, Machado S (2015) Neural mechanisms of exercise: anti-depression, neurogenesis, and serotonin signaling. CNS Neurol Disord Drug Targets 14:1307–1311. 10.2174/1871527315666151111124402 26556077

[B74] Zhang J, Fan Y, Li Y, Zhu H, Wang L, Zhu MY (2012) Chronic social defeat up-regulates expression of the serotonin transporter in rat dorsal raphe nucleus and projection regions in a glucocorticoid-dependent manner. J Neurochem 123:1054–1068. 10.1111/jnc.12055 23061525 PMC3514584

[B75] Zhang K, Fujita Y, Hashimoto K (2018) Lack of metabolism in (R)-ketamine’s antidepressant actions in a chronic social defeat stress model. Sci Rep 8:4007. 10.1038/s41598-018-22449-9 29507385 PMC5838158

